# Dissection of cellular and molecular mechanisms of aristolochic acid-induced hepatotoxicity via single-cell transcriptomics

**DOI:** 10.1093/pcmedi/pbac023

**Published:** 2022-09-22

**Authors:** Piao Luo, Jiayun Chen, Qian Zhang, Fei Xia, Chen Wang, Yunmeng Bai, Huan Tang, Dandan Liu, Liwei Gu, Qingfeng Du, Wei Xiao, Chuanbin Yang, Jigang Wang

**Affiliations:** Department of Geriatric Medicine, Shenzhen People's Hospital (The Second Clinical Medical College, Jinan University; The First Affiliated Hospital, Southern University of Science and Technology), Shenzhen 518020, China; Artemisinin Research Center, and Institute of Chinese Materia Medica, Chinese Academy of Chinese Medical Sciences, Beijing 100700, China; School of Traditional Chinese Medicine, Southern Medical University, Guangzhou 510515, China; Artemisinin Research Center, and Institute of Chinese Materia Medica, Chinese Academy of Chinese Medical Sciences, Beijing 100700, China; Department of Geriatric Medicine, Shenzhen People's Hospital (The Second Clinical Medical College, Jinan University; The First Affiliated Hospital, Southern University of Science and Technology), Shenzhen 518020, China; Artemisinin Research Center, and Institute of Chinese Materia Medica, Chinese Academy of Chinese Medical Sciences, Beijing 100700, China; School of Traditional Chinese Medicine, Southern Medical University, Guangzhou 510515, China; Artemisinin Research Center, and Institute of Chinese Materia Medica, Chinese Academy of Chinese Medical Sciences, Beijing 100700, China; Artemisinin Research Center, and Institute of Chinese Materia Medica, Chinese Academy of Chinese Medical Sciences, Beijing 100700, China; Department of Geriatric Medicine, Shenzhen People's Hospital (The Second Clinical Medical College, Jinan University; The First Affiliated Hospital, Southern University of Science and Technology), Shenzhen 518020, China; Artemisinin Research Center, and Institute of Chinese Materia Medica, Chinese Academy of Chinese Medical Sciences, Beijing 100700, China; Artemisinin Research Center, and Institute of Chinese Materia Medica, Chinese Academy of Chinese Medical Sciences, Beijing 100700, China; Artemisinin Research Center, and Institute of Chinese Materia Medica, Chinese Academy of Chinese Medical Sciences, Beijing 100700, China; School of Traditional Chinese Medicine, Southern Medical University, Guangzhou 510515, China; Key Laboratory of Glucolipid Metabolic Disorder, Ministry of Education, Guangdong Pharmaceutical University, Guangzhou 510006, China; Department of Nephrology, Integrated Hospital of Traditional Chinese Medicine, Southern Medical University, Guangzhou 510315, China; Department of Geriatric Medicine, Shenzhen People's Hospital (The Second Clinical Medical College, Jinan University; The First Affiliated Hospital, Southern University of Science and Technology), Shenzhen 518020, China; Department of Geriatric Medicine, Shenzhen People's Hospital (The Second Clinical Medical College, Jinan University; The First Affiliated Hospital, Southern University of Science and Technology), Shenzhen 518020, China; Artemisinin Research Center, and Institute of Chinese Materia Medica, Chinese Academy of Chinese Medical Sciences, Beijing 100700, China; Center for Reproductive Medicine, Dongguan Maternal and Child Health Care Hospital, Southern Medical University, Dongguan 523125, China

**Keywords:** aristolochic acid, scRNA-seq, hepatotoxicity, proteomics

## Abstract

**Background:**

Aristolochic acids (AAs), a class of carcinogenic and mutagenic natural products from *Aristolochia* and *Asarum* plants, are well-known to be responsible for inducing nephrotoxicity and urothelial carcinoma. Recently, accumulating evidence suggests that exposure to AAs could also induce hepatotoxicity and even hepatocellular carcinoma, though the mechanisms are poorly defined.

**Methods:**

Here, we aimed to dissect the underlying cellular and molecular mechanisms of aristolochic acid I (AAI)-induced hepatotoxicity by using advanced single-cell RNA sequencing (scRNA-seq) and proteomics techniques. We established the first single-cell atlas of mouse livers in response to AAI.

**Results:**

In hepatocytes, our results indicated that AAI activated NF-κB and STAT3 signaling pathways, which may contribute to the inflammatory response and apoptosis. In liver sinusoidal endothelial cells (LSECs), AAI activated multiple oxidative stress and inflammatory associated signaling pathways and induced apoptosis. Importantly, AAI induced infiltration of cytotoxic T cells and activation of proinflammatory macrophage and neutrophil cells in the liver to produce inflammatory cytokines to aggravate inflammation.

**Conclusions:**

Collectively, our study provides novel knowledge of AAs-induced molecular characteristics of hepatotoxicity at a single-cell level and suggests future treatment options for AAs associated hepatotoxicity.

## Graphical Abstract

**Graphical Abstract:**
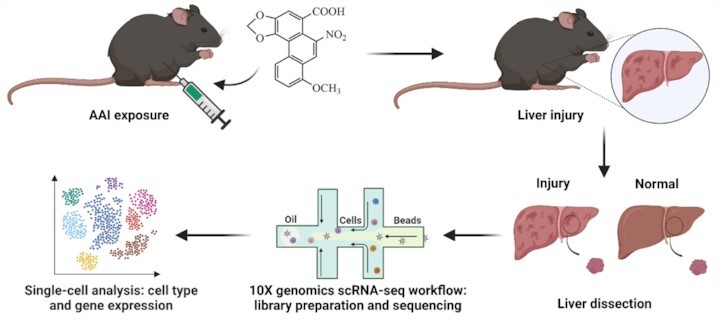
scRNA-seq analysis reveals cellular microenvironment features, clarifying the underlying mechanisms of hepatic injury in an AAI mouse model.

## Introduction

Aristolochic acids (AAs), a category of active phytochemicals commonly found in many plants such as *Aristolochia* and *Asarum*, have been used as herbal medicines worldwide for hundreds of years.^1–3^ Aristolochic acid I (AAI) is the most abundant component of AAs. Due to its toxicity, the International Agency for Research on Cancer classified AAs as the first group of human carcinogens in 2002,^4^ and many countries and regions subsequently prohibited the use of herbal medicines or preparations containing AAs.^5,6^ However, exposure to AAs is still a serious issue and people may be exposed to AAs unknowingly or be intentionally taking herbal remedies and/or food products containing AAs, or drinking groundwater that is contaminated by AAs.^7,8^ Importantly, potential health risks of AAs have been recognized as a worldwide public health issue because of the widespread circulation and improper application of herbal remedies containing AAs. It is well-known that exposure to AAs can potentially lead to various diseases, including aristolochic acid nephropathy (AAN),^9^ urothelial and gastric carcinoma,^10,11^ as well as bladder and subcutaneous cancer.^11^ Apart from nephrotoxicity, it is noteworthy that long-term overexposure to AAs induced not only hepatotoxicity^12^ but also hepatocellular carcinoma (HCC).^13^ Herbal remedies containing AAs contribute to the risk of HCC in patients with hepatitis B or C virus infection.^14,15^ Notably, in 2017, Ng *et al*. showed that AAs and their derivatives are widely implicated in liver cancers, especially in Asia,^16^ where AAs quickly became the focus of public opinion, again causing widespread concern and discussion. As such, the potential toxic mechanisms of AAs, especially their liver toxicity, have aroused the concerns of the public and researchers.

The kidney and liver are the major organs in which AAs are accumulated and metabolized.^17,18^ AAs are bioactivated into aristolactam ions that covalently bind to DNA and proteins to induce genotoxicity, carcinogenicity, and metabolic toxicity under the catalysis of endogenous cytosolic nitroreductase and microsomal enzymes.^19–23^ In addition, AAs can cause hepatic injury through oxidative stress, as well as mitochondrial apoptosis.^12^ Long-term exposure to AAs can trigger hepatic premalignant alterations via interleukin 6 recepter (IL6R)/ nuclear factor kappa-B (NF-κB) signaling activation.^24,25^ Han *et al*. found that exposure to AAs can induce liver tumorigenesis with DNA damage in mice.^13^ Although the above-proposed mechanisms partially explain AAs-induced toxicity, the cellular and molecular mechanisms of AAs-induced toxicity, especially hepatotoxicity are still not fully characterized.

Compared with bulk RNA sequence technology that determines the global transcriptome from aggregated RNA from multiple cell populations, advanced single-cell RNA sequencing (scRNA-seq) allows researchers to capture gene expression profiles at the single-cell resolution, advancing our understanding of cellular diversity and molecular function, and uncovering cellular heterogeneity and dynamic gene reprogramming.^26,27^ This methodology has been successfully employed to decipher critical pathophysiological changes including hepatoxicity,^28,29^ liver fibrosis, ^30,31^ and HCC.^32^ Therefore, scRNA-seq could provide novel insights into the molecular mechanisms of AAs-induced hepatotoxicity.

In this study, we aimed to dissect the cellular and molecular mechanisms of AAs-induced hepatoxicity at the single-cell resolution, using integrated scRNA-seq and proteomics to comprehensively identify gene expression profiles and different cell types isolated from healthy livers and AAs-treated livers. These analyses are expected to provide novel insights into our understanding of the potential mechanisms of AAs-induced hepatoxicity.

## Materials and methods

### Materials and reagents

Aristolochic acid I (AAⅠ, purity ≥98.9%) was purchased from MedChemexpress. Cell Counting Kit-8 (CCK-8) was purchased from Dojindo (Kyushu, Japan). Aspartate aminotransferase (AST) and alanine aminotransferase (ALT) kits were obtained from Bejian Xinchuangyuan Biotech (Beijing, China). Primary antibodies: anti-tumor necrosis factor α (TNF-α) (Cat# 17590–1-AP), β-actin (Cat# 66009–1-Ig), and anti-IL-1β (Cat# 16806–1-AP) were purchased from Proteintech (Chicago, USA). Anti-Bax (Cat# ab182733), anti-Bcl-2 (Cat# ab182858), anti-caspase 3 (Cat# ab184787), anti-NF-κB p65 (Cat# ab32536), anti-p-STAT3 (Cat# ab76315), and anti-CD8 (Cat# ab217344) antibodies were purchased from Abcam (Cambridge, UK).

### Cell culture and treatment

Mouse normal hepatocyte line (NCTC 1469 cell) were cultured in DMEM supplemented with 10% fetal bovine serum, penicillin and streptomycin (Gibco, Foster, CA, USA) in an adapted environment. AAⅠ was used to treat the cells for 24 or 48 h, and the cells were collected for western blotting analysis.

### Animal experiments

All animal experimental procedures were approved by the Animal Care and Use Committee of our institution. C57BL/6 mice (male, 5 weeks old) were obtained from GemPharmatech (Guangdong, China) and adapted to the environment for 1 week. Mice were randomly divided into three groups (Control, AAI-4w, AAI-8w). Mice in AAⅠ-4w group were injected intraperitoneally (i.p.) with 2 mg/kg AAI dissolved in corn oil (once a day for 4 weeks). Mice in AAⅠ-8w group were injected with 2 mg/kg AAI dissolved in corn oil by i.p. (once a day for 8 weeks). Mice in the Control group were injected with an equal volume of corn oil in the same way. Afterwards, mice were anesthetized and blood samples were collected for serum biochemical analysis. A portion of liver tissue was removed for scRNA-seq and histological analysis, the rest was stored at −80°C.

### Serum biochemical and histological analysis

Serum AST and ALT were detected by using an automatic biochemistry analyzer (TOSHIBA, Japan). Liver tissues were embedded in paraffin and cut into sections, and histological changes were assessed by hematoxylin-eosin staining (H&E) staining according to our previous study.^33^

### Western blotting analysis

Protein samples were extracted with radioimmunoprecipitation (RIPA) lysis buffer supplemented with 1 × protease inhibitor cocktail. The protein concentrations of samples were determined by bicinchoninic acid assay (BCA) kit (Thermofisher, USA). Equal amounts of proteins per group were separated by sodium dodecyl sulfate polyacrylamide gel electrophoresis (SDS-PAGE) gel, transferred to polyvinylidene fluoride (PVDF) membrane, blocked, and incubated with primary antibodies (anti-TNF-α, anti-IL-1β, anti-Bax, anti-Bcl-2, anti-caspase 3, anti-NF-κB p65, anti-p-STAT3, and anti-β-actin) overnight at 4°C, followed by incubation with the corresponding secondary antibodies. The protein bands were visualized by Azure Sapphire Analyzer and semi-quantified by ImageJ software.

### Immunofluorescence and immunohistochemistry staining

Paraffin sections of liver tissues were dewaxed and dehydrated, and then permeabilized and blocked with bovine serum albumin (BSA). Samples were then incubated with primary antibodies (anti-Bax, anti-Bcl-2, anti-CD8, anti-p-STAT3, and anti-NF-κB). For immunofluorescence (IF) staining, the corresponding fluorescent secondary antibodies and Hoechst were used. For immunohistochemistry (IHC) staining, the corresponding HRP-secondary antibodies were used. Images were acquired by a microscope (Leica TCS SP8 SR, Germany).

### Preparation of single-cell suspensions

The mice from the Control group and AAI groups exposed to 2 mg/kg/day AAI for 4 and 8 weeks were used for scRNA-seq analysis. Fresh liver tissues (three control, three AAI treatment for 4 weeks, and three AAI treatment for 8 weeks) were cut into particles and digested with a liver dissociation kit for mice (Miltenyi Biotec). According to the manufacturer's protocol, samples were filtered, centrifugated, and resuspended. Then red blood cells were removed by using a red blood cell lysis solution (Miltenyi Biotec), and washed with phosphate belanced solution (PBS) twice to generate single-cell suspensions.

### scRNA-seq

The single-cell libraries were constructed with the Single Cell 3′ Reagent Kit v3.1 10 × Genomics according to the manufacturer's instructions. Then, single-cell RNA was sequenced on an Illumina Novaseq 6000 sequencer (Illumina, San Diego, CA, USA).

### Quality control and pre-processing of the dataset

scRNA-seq raw data were subject to quality control using fastp (version 0.20.0) to organize it into high-quality data.^34^ The CellRanger count pipeline was used to generate a raw gene expression matrix [unique molecular identifier (UMI) counts per gene per cell], and analyzed by the Seurat R package in R software. Cells met the following criteria: gene number between 200 and 6000; (Rabb, #125) UMI count between 500 and 50 000; and (Rabb, #125) mitochondrial transcript detection ratio >25%. After quality control, a total of 95 955 cells (38 639 Control, 34 544 AAI-4w, and 22 772 AAI-8w) were retained and integrated into a normalized and un-batched dataset by Seurat for SCTransform function, and further subjected to principal components analysis (PCA) for dimensional reduction.

### Cell clustering and type identification

By using the Uniform Manifold Approximation and Projection for Dimension Reduction (UMAP) algorithm, cells were clustered and cell types of each cluster were identified according to the canonical markers of various cell types.

### Differentially expressed genes and pathway analysis

Differentially expressed genes (DEGs) for different cell types were analyzed by using the Seurat's FindMarkers function (|log_2_FC| ≥ 0.25, adjusted *P* value < 0.05, where FC is fold-change). DEGs were performed by heatmap and violin plots using R package's pheatmap (v1.0.12) and MySeurat Wrappers (v0.1.0).

For DEGs, Gene Ontology (GO) analysis was performed using the clusterProfiler R package^35^. Multiple hypothesis testing was corrected using the Benjamini–Hochberg procedure. Pathway analysis and activities of pathways were performed by the gene set variation analysis (GSVA) R package^36^ and Limma R package.^37^ Results with adjusted *P* value < 0.05 and |FC| > 1 were visualized.

### Pseudotime analysis

Pseudotemporal analysis was performed by using the Monocle2 R package (version 2.20.0)^38^ to reveal the cell-state transitions of CD8^+^ CTL, the transdifferentiation of cholangiocytes into hepatocytes and neutrophil cells. DEGs in each subtype were used to evaluate the differential cell states. Plots of the linage trajectories were visualized by the plot_cell trajectory_function. For neutrophil cells, branches that appeared in trajectory were analyzed by branched expression analysis modeling (BEAM) in Monocle2 to discover DEGs and DEGs were visualized by the function plot_genes_branched_heatmap.

### Cell–cell intercellular networks

Cellular communication analysis was carried out using CellphoneDB (v2.1.4)^39^ based on the ligand–receptor interactions in different cell types. Firstly, the normalized genes expression matrix and major cell types of AAI-4w, AAI-8w, and Control groups acted as input for CellphoneDB. The function method statistical_analysis was used to calculate the different numbers of pairs in AAI-4w, AAI-8w, and Control groups. The function rechart in R package recharts (v0.2–1) was used to perform data visualization. We manually selected ligand–receptor pairs which differentially expressed in AAI-4w, AAI-8w, and Control groups. Data were displayed as bubble plots using the netVisual_bubble function.

### Proteomics and data analysis

Liver tissues were lysed with RIPA lysis buffer, followed by ultrasonication on ice. Samples were centrifuged (12 000 g for 15 min at 4°C) and the supernatant was reduced and alkylated by dithiothreitol and iodoacetamide. Then samples were precipitated with precooled acetone at −20°C for at least 1 h, and then centrifuged (12 000 g for 15 min at 4°C) and the precipitate was collected and dried, and the pellet was dissolved in dissolution buffer (8 M Urea, 100 mM triethylamonium bicarbonat (TEAB), pH 8.5). Each protein sample was digested with trypsin (12.5 ng/μl) and CaCl_2_ (1 mM) at 37°C overnight and centrifuged (12 000 g for 5 min at 4°C). The supernatant was slowly loaded onto the C18 desalting column, washed with 0.1% formic acid washing buffer, and then elution buffer (0.1% formic acid, 60% acetonitrile) was added. The eluents of each sample were collected and lyophilized. Samples were then analyzed by liquid chromatography-mass spectrometry (LC-MS/MS) (Thermo Orbitrap Fusion Lumos, USA).

MS raw files were processed with Proteome Discoverer 2.4 (Thermo Scientific) and then subjected to statistical analysis and visualization in R. Differentially expressed proteins (DEPs) analysis was performed using the “limma” R package (version 3.48.3). The proteins with absolute FC ≥ 2 and adjusted *P* value [false discovery rate (FDR)] < 0.05 were defined to be DEGs. GO analysis was performed using the “clusterprofiler” R package (version 3.18.1). DEGs were visualized by Volcano plot by ggplot2 R package (version 3.3.5) based on log_2_(FC) and −log_10_(FDR) of proteins.

### Statistical analysis

All data were reported as mean ± standard error of the mean (SEM) from at least three biological replicates. GraphPad Prism 8.0 was used for statistical analyses. Ordinary one-way analysis of variance (ANOVA) was used to analyze the statistical differences between three groups of animal experimental data and two group differences were assessed for significance using Student's t test, unless otherwise mentioned. *P* value < 0.05 was considered statistically significant.

## Results

### AAI induces hepatotoxicity in mice

**Figure 1.**
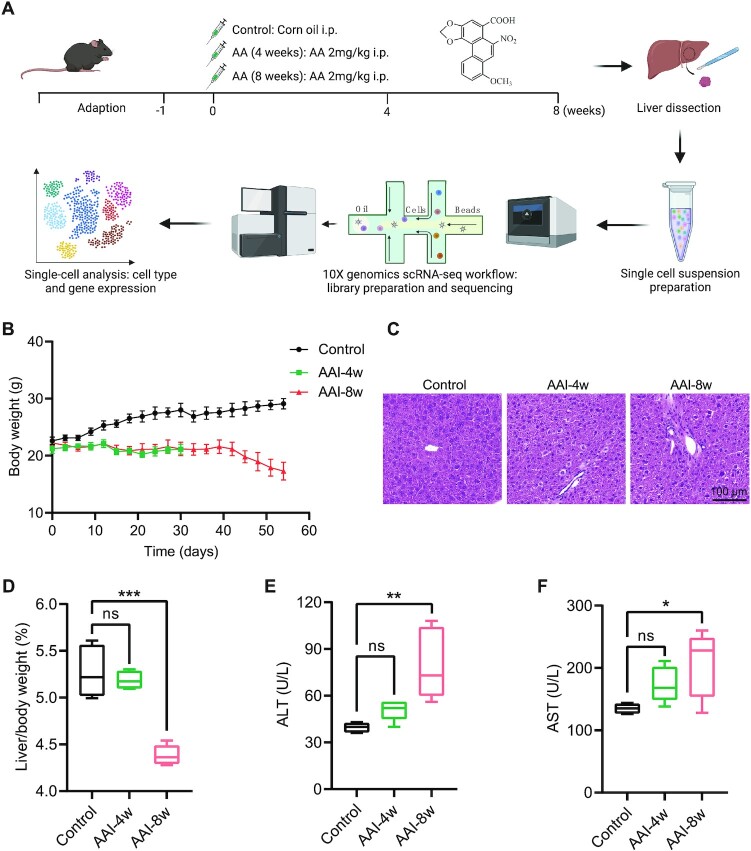
AAI induced hepatotoxicity and liver dysfunction in mice. (**A**) Experimental scheme and workflow diagram in this study. (**B**) Body weight of mice in Control, AAI-4w, and AAI-8w groups. (**C**) H&E staining showed pathological changes of mice livers after AAI treatment, scale bar = 100 μm. (**D**) Liver/body weight ratio after AAI treatment (*n* = 5, ***P < 0.001, ns = not significant). Effects of AAI on the levels of serum ALT (**E**) and AST (**F**) in the indicated groups (*n* = 5, *P < 0.05, **P < 0.01, ns = not significant).

To determine AAI-induced toxicity, we first established an AAI-induced hepatotoxicity mouse model after treatment with AAI for 4 weeks and 8 weeks, and then we used scRNA-seq to generate datasets from Control and AAI-treated mouse liver tissues to further reveal the molecular mechanisms of AAI-induced toxicity (Fig. 1A). Compared with Control mice, AAI-treated mice displayed significant signs of severe toxicity as indicated by a reduction in body weight (Fig. 1B) especially after 8 weeks AAI treatment. The H&E staining represented liver injury and morphological changes after exposure to AAI, as evidenced by the increase of hemorrhagic necrosis and inflammatory cell infiltration after 8 weeks AAI treatment (Fig. 1C). The ratio of liver weight to body weight in mice was significantly reduced after exposure to AAI for 8 weeks (Fig. 1D). Moreover, AAI increased the levels of ALT and AST, and the difference became statistically significant in the mice treated with 8 weeks of AAI (Fig. 1E and F). Our results showed that AAI induced obvious liver toxicity after 8 weeks but not after 4 weeks treatment compared with Control, which is consistent with a previous report.^40^ In summary, exposure to AAI (8 weeks) induced more severe hepatotoxicity than exposure to AAI (4 weeks) in mice.

### Identification of altered hepatic gene expression pattern via scRNA-seq and proteomics

**Figure 2.**
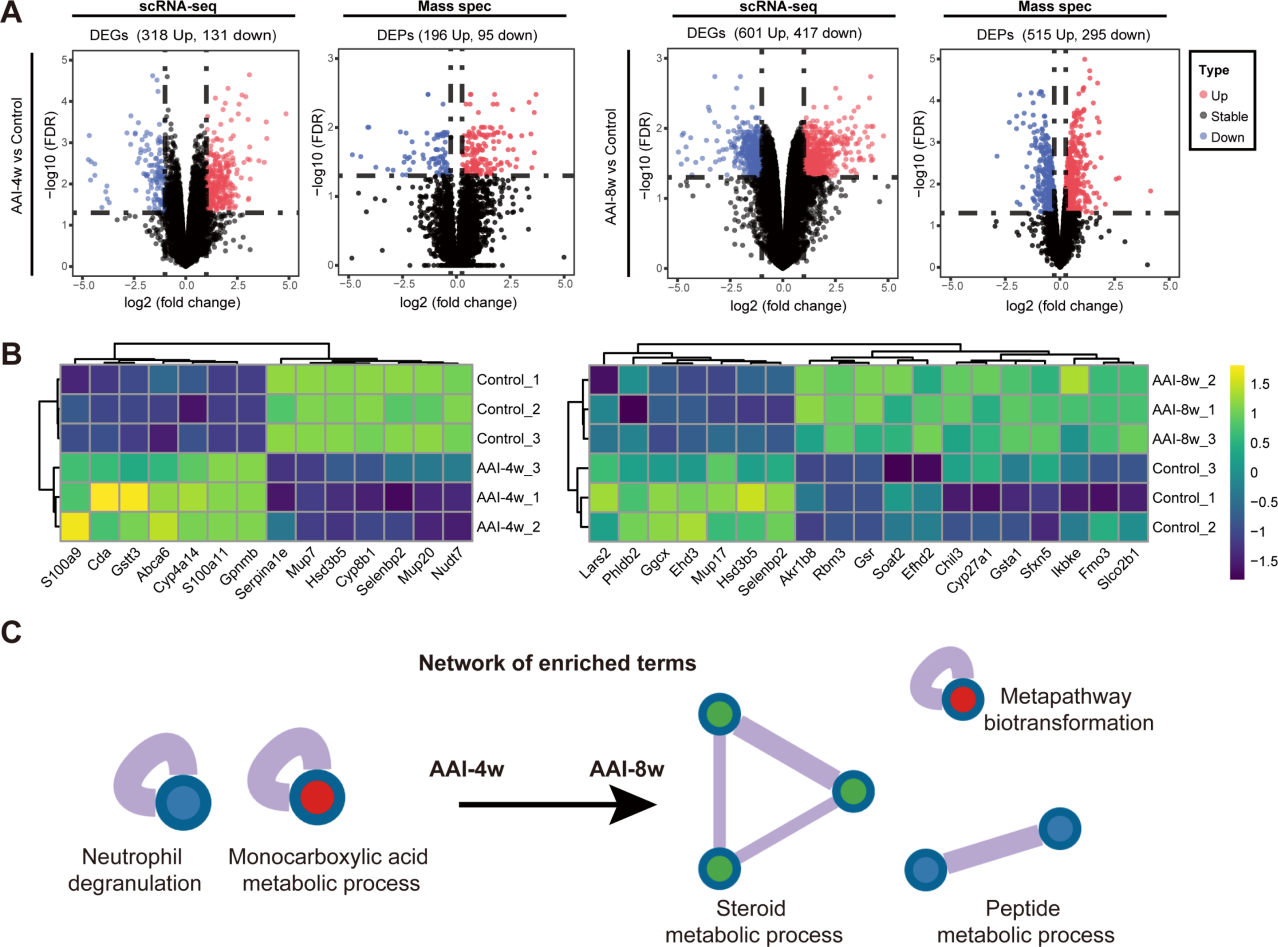
Identification of altered hepatic gene expression and functional pathways in response to AAI. (**A**) Volcano plots show DEGs and DEPs in scRNA-seq and proteomics datasets, AAI-4w *vs* Control and AAI-8w *vs* Control, respectively. (**B**) The heatmap plot indicates the relative expressions of DEGs and DEPs across two datasets. (**C**) Pathway and process enrichment analysis of DEGs and DEPs across two datasets in response to AAI.

To identify the changes in gene expression patterns as well as enriched pathways in AAI-induced liver shared by multi-omics datasets (scRNA-seq and proteomics), we first generated an *in silico* bulk RNA-seq dataset from the scRNA-seq dataset by summing raw gene counts of all cells of each sample.^41^ In the *in silico* bulk RNA-seq datasets, DEGs analysis revealed a total of 449 DEGs in AAI-4w *vs* Control (318 up, 131 down) and 1018 DEGs in AAI-8w *vs* Control (601 up, 417 down) (|FC| ≥ 2, FDR <0.05) (Fig. 2A). For DEPs, |FC| ≥ 1.2, FDR < 0.05), we quantified 4891 proteins in total, and identified 291 DEPs in AAI-4w *vs* Control (196 up, 95 down) and 810 DEPs in AAI-8w *vs* Control (515 up, 295 down) in the dataset (Fig. 2A).

Moreover, the relative expressions of DEGs and DEPs is indicated by a heatmap plot (Fig. 2B). The pathway and process enrichment analysis of these common DEGs/DEPs are associated with injury responses (Fig. 2C), such as neutrophil degranulation and the monocarboxylic acid metabolic process in the AAI-4w group, and the steroid metabolic process, metapathway biotransformation, and peptide metabolic process in the AAI-8w group. These results highlight critical roles of these pathways for AAI-induced liver injury. For instance, the induction of inflammation by neutrophil degranulation^42^ disrupted multiple metabolic pathways induced by AAI, as described in previous studies.^12,22,40^

### Single-cell transcriptomic profiles of various cell types

We further investigated the cellular and molecular mechanisms of AAI-induced hepatotoxicity in scRNA-seq datasets. A total of 103 202 cells were obtained and sequenced from three Control and six AAI-treated mouse livers, consisting of three tissue samples in each of AAI-4w and AAI-8w. After quality control, a total of 95 955 cells (38 639 Control, 34 544 AAI-4w, and 22 772 AAI-8w) (Supplementary Fig. S1, see online supplementary material) were retained and integrated into a normalized and un-batched dataset, and further subjected to PCA for dimensional reduction.

**Figure 3.**
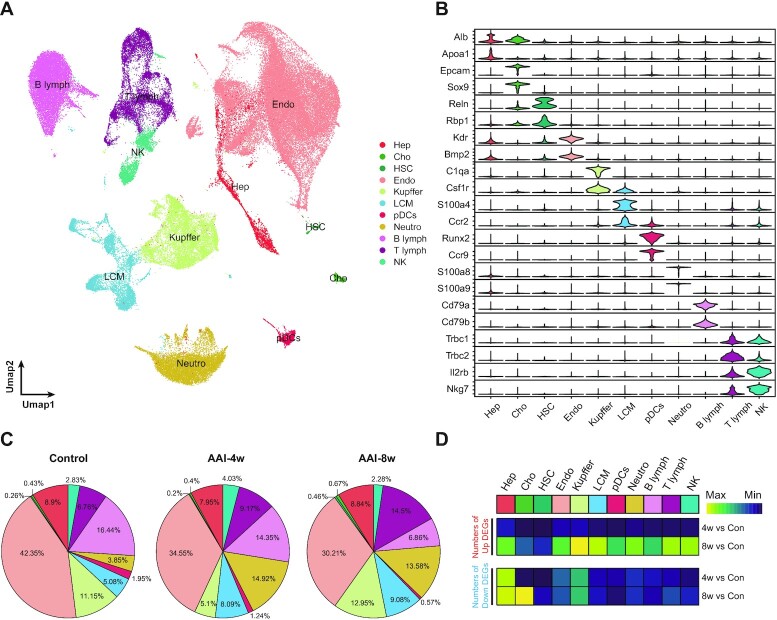
Construction of a single-cell transcriptomic atlas in mice livers in response to AAI. (**A**) Unsupervised clustering, revealing 11 distinct cellular identities by UMAP visualization. (**B**) Expression levels of selected marker genes across 11 clusters as shown by violin plot. (**C, D**) The proportion of cellular identities across three groups by pie chart according to cellular types. The relative expression of DEGs of AAI-4w (4w) or AAI-8w (8w) *vs* Control (Con) (Rabb, #125) by heatmap plot across 11 cellular types, divided into up-regulated (upper panel) and down-regulated (lower panel) DEGs.

As visualized in UMAP, our scRNA-seq dataset was resolved into 42 distinctive clusters (Supplementary Fig. S2, see online supplementary material). We then classified these cells into 11 major cell types based on the relative expressions of canonical markers in mice livers^26^ (Fig. 3A and B, and Supplementary Fig. S3, see online supplementary material). These cell types were hepatocytes (Hep), cholangiocytes (Cho), hepatic stellate cells (HSC), endothelial cells (Endo), Kupffer cells (Kupffer), liver capsule macrophages (LCM), plasmacytoid dendritic cells (pDCs), neutrophils (Neutro), B lymphocytes (B lymph), T lymphocytes (T lymph), and natural killer cells (NK).

In contrast to the control group, AAI-treated mice showed nearly 7.8%–12.14% reduction in the abundance of Endo cells, while acquiring a significantly higher fraction of immune cells such as T lymph, Neutro, and LCM cells (Fig. 3C), although obvious changes in the abundance of Hep cells were not observed in both AAI-4w and AAI-8w groups. Compared with the AAI-4w group, the number of Kupffer and T lymph of mice in the AAI-8w group showed a significant increase, while the number of NK and B lymph showed a decreasing trend (Fig. 3C). In addition, the shift in the proportion of Endo cells and immune cells indicated that AAI exposure may induce endothelial injury, accompanied by immune infiltration, which is consistent with previous reports.^13,40^ Finally, we performed DEGs analysis between AAI-4w *vs* Control and AAI-8w *vs* Control across 11 cell types (Fig. 3D). The number of DEGs in AAI-8w was much higher compared to that in AAI-4w. Overall, we have constructed a high-resolution single cell atlas of mouse livers after exposure to AAI.

### AAI induces hepatocyte apoptosis via inflammatory response

**Figure 4.**
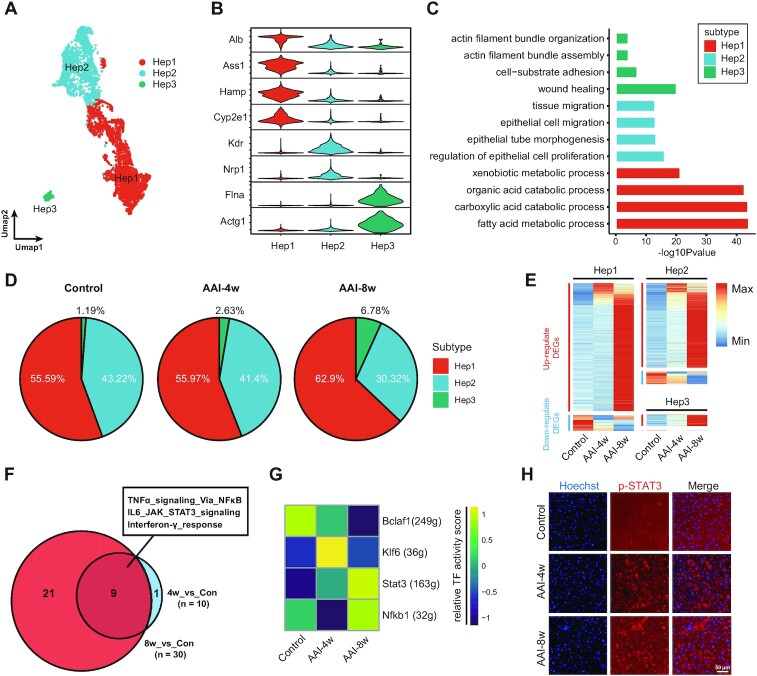
AAI induces hepatocyte cells apoptosis via promoting inflammatory response. (**A**) UMAP visualization shows unsupervised clustering of hepatocytes. (**B**) Violin plot shows the expression levels of selected marker genes across three Hep subtypes. (**C**) Bar plot depicts the top four up-regulated GO enrichments (biological process) in each Hep subtype. (**D**) Pie charts show the proportion of Hep subtypes in different groups. (**E**) Heatmap plot shows the relative expression of DEGs of AAI-4w or AAI-8w *vs* Control groups across three Hep subtypes, divided into up-regulated (upper panel, red bar) and down-regulated (lower panel, blue bar) DEGs. (**F**) GSVA analysis indicates nine overlapping up-regulated pathways in AAI-4w or AAI-8w *vs* Control groups. (**G**) Single-cell regulatory network inference and clustering (SCENIC) revealed the activities of the transcription factors. (**H**) IF staining of p-STAT3 (red) in the three groups, and the nuclei were stained by Hoechst (blue), scale bar = 50 µm.

As the compositional and functional units of the liver, hepatocytes are involved in the activation and metabolism of AAI and are considered to be a key target cell type in AAI-induced liver injury. Therefore, we first focused our analysis on hepatocyte-associated molecular changes upon AAI treatment. Unsupervised sub-clustering analysis revealed 15 subclusters from a total of 4186 Hep cells (Fig. 4A) that were further categorized into three major subtypes based on marker genes’ expression: Hep1 (highly expressed *Alb, Ass1, Hamp* and *Cyp2e1*), Hep2 (highly expressed *Kdr* and *Nrp1*), and Hep3 (highly expressed *Flna* and *Actg1*) (Fig. 4B). Interestingly, we found that Hep1 subtypes constituted the largest proportion (57.2%) of Hep cells and expressed spatially resolved marker genes along with hepatocyte zonation (Fig. 4A and B).

The functional enrichment analysis suggested the Hep1 subtypes were mainly involved in metabolic pathways such as organic acid catabolic processes and fatty acid metabolic processes, and Hep2 subtypes mainly participated in biological processes such as epithelial cell migration and the regulation of epithelial cell proliferation. Remarkably, we found that the Hep3 subtypes were associated with cell–substrate adhesion and wound healing, indicating potential cellular regeneration and injury repair functions of Hep3 subtype cells (Fig. 4C). Notably, our scRNA-seq results showed that the cellular proportion of Hep3 subtype cells increased from 1.19% (Control) to 2.63% (AAI-4w) and 6.78% (AAI-8w), which may indicate that the regenerative capabilities of hepatocytes were strengthened following AAI treatment (Fig. 4D).

Furthermore, we performed DEG analysis between the AAI-4w *vs* Control and AAI-8w *vs* Control, respectively. We found that the numbers of both up- and down-regulated DEGs significantly increased across all three Hep subtypes in AAI-8w. For instance, Hep1 showed 215 up-regulated genes for AAI-4w *vs* Control and 1193 DEGs for AAI-8w *vs* Control. The shift in the number of DEGs in Hep cells at different stages reflects the increasing toxicity effects of AAI, which was in agreement with the DEGs results (Fig. 3D).

The GSVA analysis indicated nine overlapping up-regulated pathways such as NF-κB/TNF-α signaling and STAT3/JAK/IL-6 signaling between the AAI-4w *vs* Control and the AAI-8w *vs* Control (Fig. 4F). In addition, SCENIC was used to investigate the gene regulatory networks governing the hepatocytes in response to AAI. The expression transcription factors (TFs) of STAT3 (encodes signal transduction and transcriptional activator 3, STAT3) and Nfkb1 (encodes Nuclear Factor Kappa B Subunit 1) were increased in the AAI-8w groups (Fig. 4G). Both gene set enrichment and gene regulatory networks analysis indicated that the inflammation response via STAT3 and NF-κB signaling might be activated in hepatocytes after AAI treatment. Western blotting results indicated that AAI enhanced STAT3 phosphorylation and upregulated NF-κB p65 expression in the liver of AAI-induced mice and hepatocytes induced by AAI, suggesting that AAI activated both STAT3 and NF-κB signaling *in vivo* and *in vitro* (Supplementary Fig. S4, see online supplementary material). Consistently, IF staining also showed increased phosphorylated STAT3 in hepatocytes after AAI treatment (Fig. 4H). In addition, AAI exposure also contributed to NF-κB p65 translocation into the nucleus, indicating activation of the NF-κB signaling pathway (Supplementary Fig. S5A, see online supplementary material), which may then up-regulate the expression of TNF-α in hepatocyte cells (Supplementary Fig. S5B). Immunohistochemistry staining showed that AAI exposure up-regulated the expression of caspase 3, a canonical apoptosis marker (Supplementary Fig. S5C). These results suggest that hepatocytes display an inflammatory effect mediated by the STAT3/NF-κB signaling pathway after AAI treatment, which may initiate the expression of the apoptotic factor caspase 3 and contribute to cell death.

To investigate other molecular mechanisms of hepatic regeneration and repair, we reconstructed the developmental trajectory of the transdifferentiation of cholangiocytes into hepatocytes, revealing that cholangiocytes could differentiate into hepatocytes, with the latter compensating for or repairing hepatocyte damaged by AAI (Supplementary Fig. S6A, see online supplementary material). To further explore the molecular mechanisms underlying the different trajectories, we retrieved dynamic gene expression change as differentiation progresses and performed biological progress enrichment (Supplementary Fig. S6B). The results suggested that cells (State 1) were enriched for the genes involved in response to wounding and muscle tissue development, cells (State 2) were enriched for the genes involved in notch signaling, wounding healing and vasculature development, while hepatocytes (State 3) were enriched for the genes involved in metabolic processes.

### Endothelial cells exhibit apoptotic response to AAI

**Figure 5.**
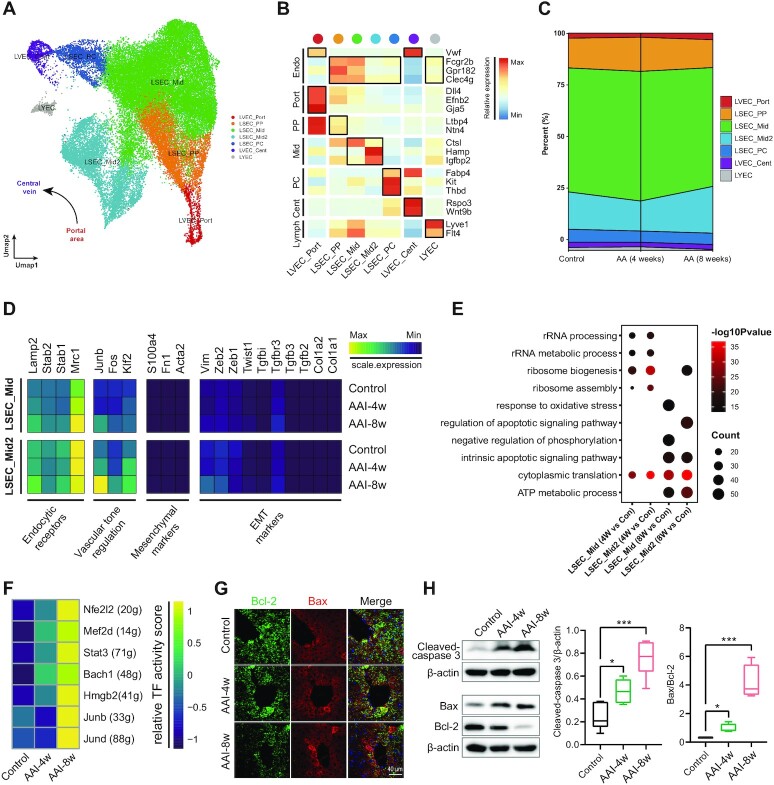
Endothelial cells exhibit apoptotic response to AAI. (**A**) UMAP visualization shows unsupervised clustering, revealing seven distinct subtypes of endothelial cells. (**B**) Heatmap plot depicts the cellular marker genes' expression in each subtype of the endothelial cells subpopulation. (**C**) Cumulative area plot reveals the relative proportion of each endothelial cells subpopulation in Control, AAI-4w, and AAI-8w, respectively. (**D**) Heatmap plot shows the expression levels of pathways-associated genes in Control, AAI-4w, and AAI-8w groups with LSEC_Mid (upper panel) and LSEC_Mid2 (lower panel) cellular subtypes. (**E**) Bubble plot shows the GO enrichment pathways of up-regulated genes in LSEC_Mid and LSEC_Mid2 subtypes upon AAI treatment. (**F**) SCENIC revealed the increases of the transcription factors expression in endothelial cells after AAI treatment. (**G**) IF staining of Bcl-2 (green) and Bax (Red) to detect endothelial cell apoptosis in the three groups, the nucleus was labelled by Hoechst (blue), scale bar = 40 µm. (**H**) Western blot was used to detect the expression of apoptotic signaling pathway-related markers cleaved-caspase 3, Bax, and Bcl-2 in liver tissues after AAI treatment (*n* = 4; *P < 0.05, ***P < 0.001 vs Control).

The hepatic vasculature is broadly compartmentalized into the portal vein, hepatic artery, central vein, and sinusoids in the liver, with the endothelial cells located across different zones of the liver with specific gene expression patterns.^26^ According to the spatial lobular locations and functional markers^41^ of portal (port), periportal (pp), middle (Mid), pericentral (PC), central (Cent), and lymphatic, we divided 39 190 Endo cells into four groups of liver sinusoidal endothelial cells (LSECs), two groups of vascular endothelial cells (LVECs), and one group of lymphatic endothelial cell (LYESs) (Fig. 5A and B).

The LSEC_mid and LSEC_mid2 subtypes exhibited the most significant proportional changes after AAI treatment. The cellular proportions of the LSEC_Mid subtype were 57.07%, 59.67%, and 54.74% in Control, AAI-4w, and AAI-8w groups, respectively. For the LSEC_Mid2 subtype, the proportions were 17.2%, 13.82%, and 21.49% in Control, AAI-4w, and AAI-8w groups, respectively (Fig. 5C). To determine alterations in the classical endothelial functional pathway after AAI treatment, we investigated the marker gene expression of endocytic receptors, vascular tone regulation, mesenchymal, and EMT pathways of two LSEC_Mid subtypes after AAI treatment. Although obvious alterations in gene expression of most of these pathways were not observed in these two subtypes, we found increased *Junb* and *Klf2* expression in LSEC_Mid2 of the AAI-8w group, indicating that AAI treatment might trigger endothelial response via the vascular tone regulation pathway (Fig. 5D).

Furthermore, we performed GO enrichment analysis based on up-regulated genes between AAI treatment and control groups in LSEC_Mid and LSEC_Mid2 cells. In the five top biological processes of the AAI-4w group, both subtypes showed up-regulation of the ribosome-associated pathways such as ribosome biogenesis and rRNA processing. However, we found that both of these two subtypes up-regulated the intrinsic apoptotic signaling pathway after AAI treatment for 8 weeks. Specifically, the LSEC_Mid subtype activated the response to the oxidative stress pathway and activated the regulation of apoptotic signaling pathway (Fig. 5E). Therefore, we examined the expression of apoptotic markers, such as Bax, Bcl-2 and cleaved-caspase 3, by immunostaining or western blotting analysis. Our results showed that AAI exposure up-regulated pro-apoptotic Bax expression and down-regulated anti-apoptotic Bcl-2 expression, and increased caspase 3 (Fig. 5G and H). These results indicate that AAI induced apoptosis in endothelial cells especially for LSEC subtypes. In addition, compared with the Control and AAI-4w groups, the SCENIC results indicate that endothelial cells had the same activation of STAT3 as hepatocytes in the AAI-8w group. There are several TFs such as Nfe2l2 (encodes nuclear factor E2 related factor 2, Nrf2), Bach1 (encodes BTB Domain and CNC Homolog 1) activated in the AAI-8w group (Fig. 5F). Most of these TFs are involved in oxidative stress regulation during liver injury as reported in previous studies,^43^ suggesting a potential role of oxidative stress in AAI-induced endothelial apoptosis.

### AAI induces robust liver infiltration of cytotoxic T cells

**Figure 6.**
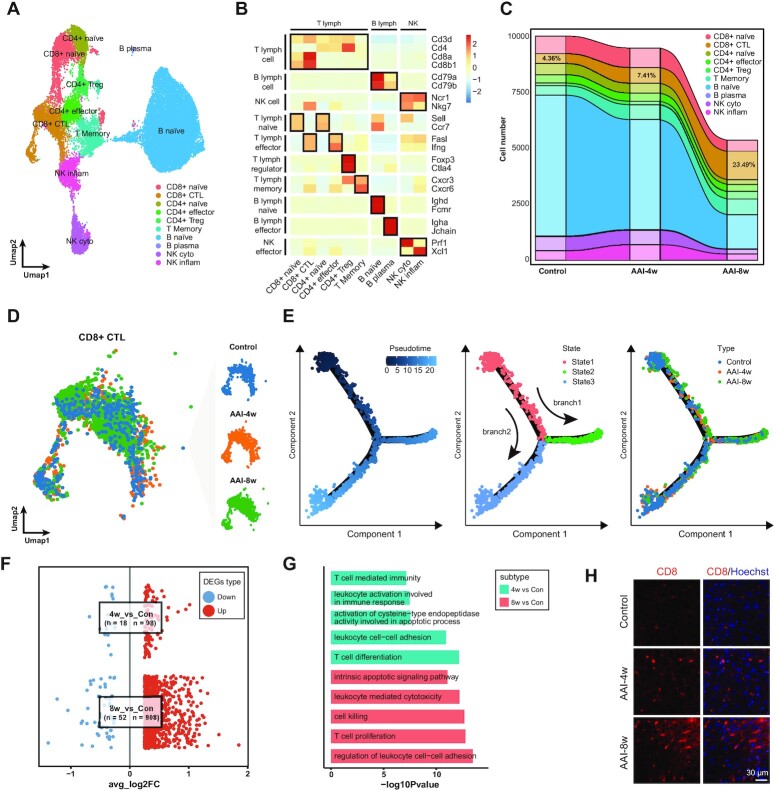
AAI induces robust liver infiltration of cytotoxic T cells. (**A**) UMAP visualization shows unsupervised clustering, revealing 10 distinct subtypes of lymphocytes. (**B**) Heatmap plot depicts the cellular marker genes’ expression in each subtype in B lymphocyte, T lymphocyte, and NK cells. (**C**) Sankey plot reveals the relative proportion change of each lymphocyte cell subpopulation in Control, AAI-4w, and AAI-8w, respectively. (**D**) UMAP visualization shows the unsupervised clustering of CD8^+^ CTL, and split into three subclusters: Control, AAI-4w, and AAI-8w, respectively. (**E**) Monocle trajectory inference of CD8^+^ CTL cells traces a path of pesudotime (left), and labeled with the cell state (middle), and group types (right), respectively. (**F**) Visualization shows the scatter plot of average log_2_FC values for both up-regulated and down-regulated genes in AAI-4w (4w) *vs* Control (Con) (Rabb, #125) and AAI-8w (8w) *vs* Control (Rabb, #125). (**G**) Bar plot depicts the top five up-regulated GO enrichment (biological process) of CD8^+^ CTL subtype in AAI-4w (4w) *vs* Control (Rabb, #125) and AAI-8w (8w) *vs* Control (Rabb, #125). (**H**) IF staining of CD8^+^ CTL in liver tissues after AAI treatment, scale bar = 30 µm.

The lymphocyte population, including T lymphocytes, B lymphocytes and NK cells, plays important roles in maintaining homeostasis and immune response in the liver. Here, we re-clustered and further categorized 24 961 lymphocytes into 10 subtypes based on their marker genes’ expression (Fig. 6A). These cell types include CD8^+^ T naïve cells (CD8^+^ naïve; *Cd8*^+^Sell^+^*Ccr7*^+^), CD8^+^ cytotoxic T cells (CD8^+^ CTL; *Cd8*^+^*Fasl*^+^*Ifng*^+^), CD4^+^ T naïve cells (CD4^+^ naïve; *Cd4*^+^*Sell*^+^*Ccr7*^+^), CD4^+^ T effector cells (CD4^+^Te; *Cd4*^+^*Fasl*^+^*Ifng*^+^), CD4^+^ T regulatory cells (CD4^+^Treg; *Cd4*^+^*Foxp3*^+^*Ctla4*^+^), T memory cells (T Memory; *Cd4*^+^*Cxcr3*^+^), B naïve cells (B naïve; *Cd79a*^+^*Ighd*^+^*Fcmr*^+^), B plasma cells (B plasma; *Cd79a*^+^*Igha*^+^*Jchain*^+^), NK cytotoxic cells (NK cyto; *Ncr1*^+^*Prf1*^+^), and NK inflammatory cells (NK inflam; *Ncr1*^+^*Xcl1*^+^) (Fig. 6B). As shown in the Sankey plot, among the 10 lymphocyte subtypes, we noticed a gradually increasing cellular proportion of CD8^+^ CTL as the AAI treatment period extended, ranging from 4.36% (Control) to 7.41% (AAI-4w) and ultimately to 23.49% (AAI-8w). These results highlight a critical role of CD8^+^ CTL in AAI-induced liver injury (Fig. 6C).

To further determine the status changes and gene expression patterns of CD8^+^ CTL after AAI treatment, we extracted the CD8^+^ CTL subtype from the whole lymphocyte population (Fig. 6D). The cell trajectory results revealed that as the pseudotime passed, the CD8^+^ CTL subtype was clearly divided into two main branches: branch1 (from State1 to State2) and branch2 (from State1 to State3). We found that CD8^+^ CTL cells of the AAI-8w group were mainly distributed at branch2 of psudotime, while CD8^+^ CTL cells from the other two groups were distributed at branch1 of psudotime, indicating the differential state of CD8^+^ CTL cell in AAI-8w group. In addition, DEGs were identified in the CD8^+^ CTL subtype after AAI treatment (Fig. 6E). Similar to the other cell types, the number of AAI-8w *vs* Control DEGs (908 up-regulated and 52 down-regulated) was much more than that of AAI-4w *vs* Control DEGs (98 up-regulated and 18 down-regulated) (Fig. 6F). The enrichment analysis indicated that CD8^+^ CTL cells in the AAI-4w group up-regulated pathways including leukocyte cell–cell adhesion and T cell differentiation, while those in the AAI-8w group were involved in the intrinsic apoptotic signaling pathway, leukocyte mediated cytotoxicity, and cell killing. These results indicated that AAI induces robust liver infiltration of cytotoxic T cells (Fig. 6G). The recruitment of CD8^+^ CTL cells after AAI treatment was further confirmed by immunostaining (Fig. 6H). The increased CD8^+^ CTL cells may be associated with increases in hepatic inflammation, fibrosis, and nonalcoholic steatohepatitis.^44,45^

### AAI treatment induces an inflammatory response in macrophages and neutrophils

**Figure 7.**
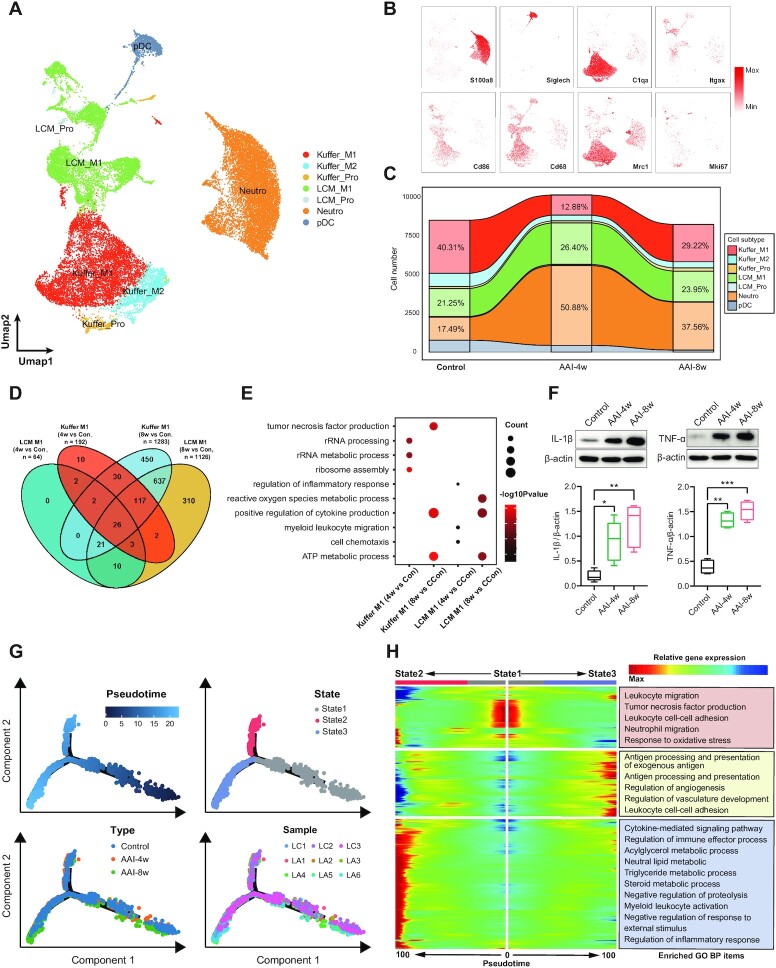
Activated macrophage and neutrophil cells aggravate inflammatory response induced by AAI. (**A**) UMAP visualization shows unsupervised clustering, revealing seven distinct subtypes of Kupffer, LCM, Neutro, and pDC cell. (**B**) UMAP plot shows representative cellular marker genes’ expression levels in the indicated myeloid cells. (**C**) Sankey plot reveals the relative proportion change of each myeloid cells subpopulation in Control, AAI-4w, and AAI-8w, respectively. (**D**) Venn plot shows overlapping DEGs in 4w *vs* C and 8w *vs* C of Kupffer_M1 and LCM_M1 subtypes; 4w, AAI-4w; 8w, AAI-8w; C, Control. (**E**) Bubble plot shows the GO enrichment biological process (BP) items of 4w *vs* C and 8w *vs* C for up-regulated DEGs in Kuffer_M1 and LCM_M1 subtypes; 4w, AAI-4w; 8w, AAI-8w; C, Control. (**F**) Western blot showing the expression of inflammatory cytokines IL-1β and TNF-α in liver after AAI treatment (*n* = 4, *P < 0.05, **P < 0.01, ***P < 0.001 vs Control). (**G**) Monocle trajectory inference of neutrophil cells traces the path of pesudotime (top left), and labeled with the cell state (top right), group types (bottom left), and sample ID (bottom right), respectively. (**H**) Heatmap plot reveals the relative gene expression level of clusters at two branches (state1 to state2 and state1 to state3) based on branched expression analysis modeling (right), combined with the up-regulated GO enriched items of each cluster (left) after AAI treatment.

Myeloid cells in liver mainly consist of tissue-residual Kupffer macrophages, liver capsular macrophages (LCMs), plasmacytoid dendritic cells (pDC), and Neutro. These cell types have heterogeneous distributions and can self-replenish in response to drug-induced liver injury.^46^ Here, LCMs and Kupffer cells were further divided into three subtypes named M1 (expressing *Cd86, Cd68*), M2 (expressing *Mrc1*), and proliferation (Pro, expressing *Mki67*) subtypes (Fig. 7A and B). M1-like macrophages are mainly involved in pro-inflammatory responses.^47^ Interestingly, dramatic changes in the proportion of Kuffer_M1, LCM_M1, and Neutro were observed after AAI treatment (Fig. 7C), indicating the increased pro-inflammatory effects in these cells in response to AAI treatment.

Moreover, after AAI treatment for 4 weeks, 64 and 192 up-regulated genes were identified in LCM_M1 and Kuffer_M1 subtypes, respectively. In the AAI-8w group, the DEGs numbers increased to 1126 and 1283 respectively. A total of 26 overlapping up-regulated DEGs were observed in all four macrophage subtypes, while there were 801 overlapping up-regulated DEGs between LCM_M1 (AAI-8w *vs*. Control) and Kuffer_M1 (AAI-8w *vs* Control), revealing that the AAI treatment induced similar gene alterations to these two subtypes after 8 weeks (Fig. 7D). Furthermore, the GO enrichment indicated that Kupffer M1 subtype cells in the AAI-4w group activated pathways including rRNA processing and ribosome assembly, while LCM_M1 in the AAI-4w group activated pathways associated with the regulation of inflammatory response and cell chemotaxis. Meanwhile, these two subtypes in the AAI-8w group were involved in the positive regulation of cytokine production, reactive oxygen species metabolic processes, and tumor necrosis factor production (Fig. 7E). Increased expression levels of pro-inflammatory cytokines such as IL-1β and TNF-α were validated by western blot assay, indicating an activated inflammatory response in macrophage subtypes after AAI treatment (Fig. 7F).

Lastly, we constructed the lineage of Neutro cells to investigate cellular state changes and biological processes regulation. As shown in Fig. 7G, the lineage revealed two branches (branch1: from state1 to state2; branch2: from state1 to state3) from the beginning to the end of pseudotime. We also found that Neutro cells in the AAI-8w group were mainly converted into state3 while those in AAI-4w group were located in state2, in agreement with the sample identities (LC1-3 were in the control group, LA4-6 were in the AAI-4w group and LA7-9 were in the AAI-8w group). We further performed BEAM to reveal three clusters of DEGs and their activated pathways at the branching point (Fig. 7H). In contrast to state1, Neutro cells at state2 activated pathways including response to cytokine-mediated signaling, myeloid leukocyte activation, and regulation of inflammatory response. Meanwhile, Neutro cells at state2 activated pathways including antigen processing and presentation, the regulation of vasculature development, and participated in leukocyte cell–cell adhesion, etc. Collectively, our results demonstrated that macrophages and neutrophils were specifically recruited and hyper-activated after AAI treatment for 8 weeks.

### Intercellular networks for the response to AAI

To explore the differential cell–cell interactions after AAI treatment, we constructed an intercellular network between different cell types using potential ligand–receptor pairs, including Hep, Cho, HSC, Endo, Kupffer, LCM, pDCs, Neutro, B lymph, T lymph, and NK (Supplementary Fig. S7A, see online supplementary material). We next analyzed the cross-talk variance of subtypes within differential cell types. Interactions between Hep, Cho, and other cell types are enhanced with prolonged AAI administration (Supplementary Fig. S7B). Next, the specific ligand–receptor pairs forming the interaction network are summarized (Supplementary Fig. S7C). Compared with AAI-4w, we further found an increased communication probability of TNF–TNF receptor pairs between Hep, Cho, and macrophages (Kupffer and LCM) in the AAI-8w group (Supplementary Fig. S7C). TNF isoforms, TNF-α and TNF-β, are the major known upstream signals of the NF-κB pathway. Western blotting and IF or IHC assays showed the activation of NF-κB in AAI treatment compared to the Control group (Supplementary Fig. S4 and Fig. S5). Therefore, these results support the activation of NF-κB and inflammation induced by AAI.

## Discussion

AA and its derivatives cause nephrotoxicity and urinary tract tumors, an effect that has been widely recognized in the past few decades.^23,48^ It has also been reported that long-term use of AAs-containing herbal remedies has been linked to tumors in multiple organs, including kidney, stomach, bladder, and subcutaneous tumors.^11^ Although herbal preparations containing AAs have been phased out in multiple countries such as Japan, Canada, the UK, the USA, Australia, and Europe,^5,49^ there are still some regions in which people are exposed to AAs by inadvertently taking certain herbal medicines.^6^ Moreover, AAs are a class of persistent soil pollutants, and their substantial accumulation in agricultural fields has caused serious food pollution problems and they may finally accumulate in human bodies.^8^ As such, exposure to AAs remains a worldwide concern.

**Figure 8.**
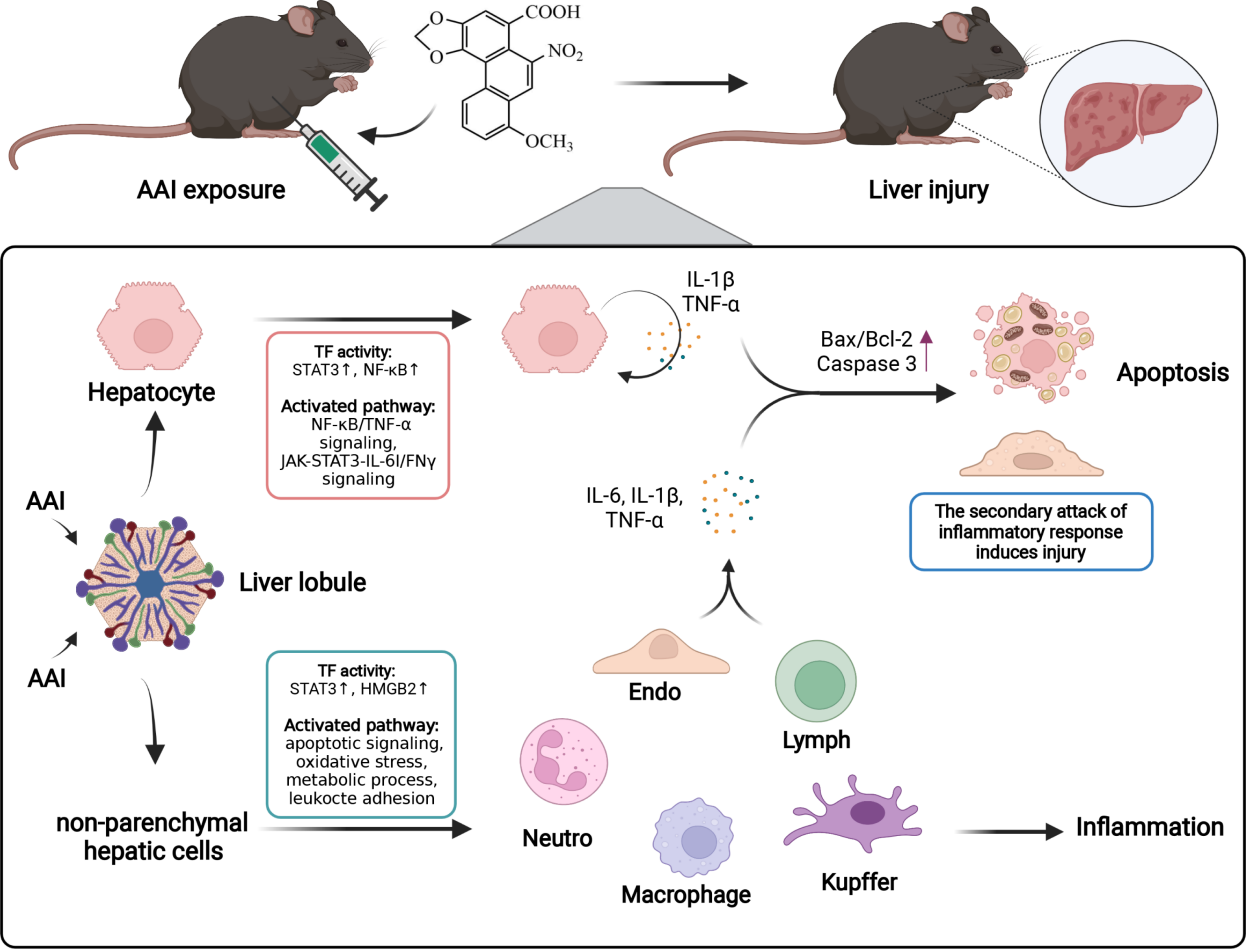
Schematic summary of the potential mechanisms of AAI-induced hepatotoxicity. First, AAI activated NF-κB and STAT3 signaling pathways contributing to inflammatory response and apoptosis, and AAI activated multiple oxidative stress and inflammatory associated signaling pathways and induced apoptosis in LSECs. Moreover, AAI induced infiltration of cytotoxic T cells, macrophages, and neutrophil cells in the liver to produce inflammatory cytokines to aggravate inflammation. In brief, the scRNA-seq analysis reveals cellular microenvironment features, clarifying the underlying mechanisms of hepatic injury and inflammation response in an AAI mouse model.

The toxic mechanisms of AAs have been studied in multiple animal models. Apart from well-established kidney toxicity, it has recently been reported that AAs could also be closely related to the occurrence and pathogenesis of liver cancer due to their genotoxicities.^13,14,16^ In this present study, for the first time, we used scRNA-seq technology to establish a high-resolution single-cell mouse liver atlas in response to AAI. As shown in the summary graphic, the molecular and cellular mechanisms of AAI-induced hepatotoxicity were reprogramed at the single-cell level (Fig. 8). Furthermore, we integrated the scRNA-seq dataset with the proteomics dataset to reveal the cellular and molecular characteristics of AAI-induced toxicity. Compared with previous studies,^40^ our data comprehensively revealed that AAI induced inflammation and cell apoptosis in liver via disrupting multiple metabolic pathways. Similarly, we previously integrated single-cell transcriptomics and proteomics to reveal immune cell-specific responses and immune microenvironment remodeling in AAN.^50^

Long-term exposure to AAI could aggravate liver injury more than the short-term exposure.^40^ This study also showed that exposure to AAI for 8 weeks caused more severe hepatotoxicity than exposure to AAI for 4 weeks in mice. Integration of single-cell transcriptomic and proteomic data revealed significant alterations of cell-type and immune microenvironment in AAI-induced liver disease with prolonged administration. The heterogeneity of hepatic cells has been revealed by scRNA-seq in mammalian livers.^51–53^ Hepatocytes are one of the most essential components in the liver for inflammatory responses. In the current study, hepatocytes were divided into three subtypes according to specific gene expression profiles after AAI treatment. In-depth analysis revealed the relationship between hepatocyte subtypes and the divergence of response to AAI treatment, further uncovering that AAI specifically increased the number of Hep3 subtype hepatocytes, which are characterized in wound healing and metabolic processes. Some studies showed that liver stem cells and Cho cells can give rise to hepatocytes for hepatic regeneration and repair in liver injury.^54,55^ Therefore, in chronic liver injury, types of cells other than hepatocytes differentiate and repair liver injury, which is worthy of attention. In this study, there is a potential mechanism for the differentiation of Cho into hepatocytes to repair AAI-induced injury. In recent years, studies have focused on the immunomodulatory functions of hepatocytes secreting inflammatory cytokines such as TNF-α and IL-6^56^. It has been reported that pro-inflammatory cytokines secreted by hepatocytes promote liver damage.^57,58^ In our scRNA-seq results, the inflammatory hepatocytes were involved in the immune-inflammatory response regulated via activating two distinct transcription factors, NF-kB and STAT3, during AAI-induced liver injury. NF-kB and STAT3 activation promoted the expression of inflammation-related genes including TNF-α and IL-6. These cytokines further exacerbated inflammation, leading to hepatocyte injury. Consistent with previous studies, NF-kB activation enhances TNF-α-mediated hepatocyte apoptosis.^59,60^ Moreover, the activation of STAT3/p-STAT3 and NF-κB/IL6R signaling participates in the regulation of AAI-induced acute hepatic premalignant alteration in canine liver.^24,25^ Overall, our data showed that the increased a subtype of hepatocytes and activated NF-κB and STAT3 signaling pathways may contribute to AAI-induced liver injury in hepatocytes.

LSECs, hepatic endothelial cells, play a key role in liver functions.^61^ LSECs can clear out circulating xenobiotics and toxins via endocytic receptors and underpin the homeostasis of hepatic sinusoids.^62,63^ We found shifts of the classical endothelial functional pathways including vascular tone regulation in the AAI-induced liver. Thus, AAI treatment could facilitate the impairment of vascular control of the LSECs, inflammation, and liver regeneration.^64^ It is known that oxidative stress and inflammation are the major factors of LSECs dysfunction.^65^ In this study, GO enrichment analysis of DEGs indicated that AAs induced oxidative stress and apoptotic signaling pathway. In addition, TFs involved in oxidative stress- and inflammation-related TFs such as Nrf2, Bach1, STAT3, and HMGB2 were activated in the AAI groups.^66,67^ Taken together, AAI treatment induces LSECs apoptosis through oxidative stress and inflammation, which may be driven by key pathways such as Nrf2 and STAT3/HMGB2 signaling.

AAs treatment contributes to the proliferation and infiltration of lymphocytes and macrophage populations in mouse models and humans.^12,68^ Our results demonstrated the heterogeneous response of lymphocytes and macrophages from mouse liver to AAI treatment. Previous studies have indicated that CD4^+^ and CD8^+^ lymphocytes are widely implicated in AA-induced renal injury.^45^ Here, we found that the proportion of CD8^+^ CTL cells gradually increased with prolongation of the AAI treatment period. DEGs from gene expression patterns were involved in leukocyte cell–cell adhesion, leukocyte-mediated cytotoxicity, and theintrinsic apoptotic signaling pathway after AAI treatment, suggesting that CD8^+^ CTL cells are closely related to liver injury caused by AAI. Macrophages, LCMs, and Kupffer cells presented an M1-skewed phenotype. M1 macrophages can secrete pro-inflammatory cytokines and contribute to the inflammatory response, leading to liver injury.^69,70^ For instance, IL-1β is one of the major pro-inflammatory cytokines triggering liver injury such as hepatic ischemia–reperfusion injury and drug-liver injury.^71,72^ TNF-α is a potent immune-inflammatory cytokine mainly secreted by immune cells, modulating liver damage.^73,74^ AAI can trigger the activation and accumulation of macrophages, and promote the production of macrophage-related factors, aggravating the injury.^75,76^ Our scRNA-seq data indicated that M1 macrophages promoted the inflammatory response and cell chemotaxis in the AAI-4w group, and increased cytokine production such as IL-1β and TNF-α and reactive oxygen species metabolic processes in the AAI-8w group. These results showed that AAI treatment induces an inflammatory response in macrophages. In addition, it has been reported that neutrophils are involved in various liver diseases including alcoholic liver disease and drug-induced liver injury.^77^ We constructed the lineage of Neutro cells to reveal the cellular state changes and biological process regulation, including cytokine-mediated inflammatory response and leukocyte cell–cell adhesion, suggesting that AAI treatment induces an inflammatory response in Neutro cells. Therefore, our results demonstrated that CTL, activated macrophages, and neutrophils were specially recruited to liver after AAI treatment, suggesting that AAI induced the inflammatory response in liver.

However, there are several limitations to this study. Firstly, we focused on relatively short- and medium-term AAI-induced hepatotoxicity relative to previous studies of induced hepatocellular carcinoma;^13^ future studies aiming to detect long-term AAI-induced liver cancer will be needed. Secondly, microenvironmental alterations associated with carcinogenesis due to AAI inducing A→T transversions will be needed. Thirdly, under the conditions of long-term induction of liver cancer by AAI and the presence of A→T transversions and AAI–DNA adducts,^20,23^ it is urgent to dissect the development of liver carcinogenesis by single-cell sequencing and multi-omics combined analysis strategies.

In summary, for the first time, this study provides insights into the underlying cellular and molecular mechanisms of AAI-induced hepatotoxicity by using scRNA-seq. It comprehensively reveals that AAI triggered an inflammatory response via NF-κB and STAT3 signaling pathways in hepatocytes and induced LSEC apoptosis via STAT3/HMGB2 signaling pathways. Moreover, AAI induced infiltration of cytotoxic T cells, and activated macrophage and neutrophil cell-induced inflammatory responses. Overall, our study suggests that AAI activates the immune-inflammatory system, thereby promoting apoptosis and resulting in hepatotoxicity. Collectively, our study provides a novel insight into the molecular characteristics of AAI-induced liver injury at a single-cell level, and provides pathways for future therapeutic intervention potential for AAI-induced liver injury.

## Supplementary Material

pbac023_Supplemental_File

## Data Availability

The raw data files were deposited in the Genome Sequence Archive (GSA) under accession number CRA006905. The R script and relative dataset files used in this study are available at https://github.com/Nino5105/AAI_liver_multi-omics_code.

## References

[1] Yang H-Yu , Chen P-C, Wang J-D. Chinese herbs containing aristolochic acid associated with renal failure and urothelial carcinoma: a review from epidemiologic observations to causal inference. Biomed Res Int. 2014;2014:569325. doi: 10.1155/2014/569325. 25431765 PMC4241283

[2] Michl J , Ingrouille MJ, Simmonds MSJ, et al. Naturally occurring aristolochic acid analogues and their toxicities. Nat Prod Rep. 2014;31:676–93.. doi: 10.1039/c3np70114j. 24691743

[3] Abdullah R , Diaz LN, Wesseling S, et al. Risk assessment of plant food supplements and other herbal products containing aristolochic acids using the margin of exposure (MOE) approach. Food Addit Contam Part A Chem Anal Control Expo Risk Assess. 2017;34:135–44.. doi: 10.1080/19440049.2016.1266098. 27892830

[4] IARC Working Group on the Evaluation of Carcinogenic Risks to Humans. Some traditional herbal medicines, some mycotoxins, naphthalene and styrene. IARC Monogr Eval Carcinog Risks Hum. 2002;82:1–556. 12687954 PMC4781602

[5] Debelle F , Vanherweghem J-L, Nortier JL. Aristolochic acid nephropathy: a worldwide problem. Kidney Int. 2008;74:158–69.. doi: 10.1038/ki.2008.129. 18418355

[6] Zhang H-M , Zhao X-Hu, Sun Z-H, et al. Recognition of the toxicity of aristolochic acid. J Clin Pharm Ther. 2019;44:157–62.. doi: 10.1111/jcpt.12789. 30548302

[7] Riffault-Valois L , Wattez C, Langrand J, et al. Health risk associated with the oral consumption of “Chiniy-tref”, a traditional medicinal preparation used in Martinique (French West Indies): Qualitative and quantitative analyses of aristolochic acids contained therein. Toxicon. 2019;172:53–60.. doi: 10.1016/j.toxicon.2019.10.241. 31704310

[8] Chan C-K , Tung Ka-Ki, Pavloviä NM, et al. Remediation of aristolochic acid-contaminated soil by an effective advanced oxidation process. Sci Total Environ. 2020;720:137528. doi: 10.1016/j.scitotenv.2020.137528. 32143041

[9] Vanherweghem J-L , Tielemans C, Abramowicz D, et al. Rapidly progressive interstitial renal fibrosis in young women: association with slimming regimen including Chinese herbs. Lancet (London, England). 1993;341:387–91.. doi: 10.1016/0140-6736(93)92984-2. 8094166

[10] Wang L , Li C, Tian J, et al. Genome-wide transcriptional analysis of Aristolochia manshuriensis induced gastric carcinoma. Pharm Biol. 2020;58:98–106.. doi: 10.1080/13880209.2019.1710219. 31957525 PMC7006638

[11] Wang L , Ding X, Li C, et al. Oral administration of Aristolochia manshuriensis Kom in rats induces tumors in multiple organs. J Ethnopharmacol. 2018;225:81–9.. doi: 10.1016/j.jep.2018.07.001. 30008395

[12] Wang Y , Ma X, Zhou C, et al. Aristolochic acid induces mitochondrial apoptosis through oxidative stress in rats, leading to liver damage. Toxicol Mech Methods. 2021;31:609–18.. doi: 10.1080/15376516.2021.1946229. 34167444

[13] Lu ZN , Luo Q, Zhao LN, et al. The mutational features of aristolochic acid-induced mouse and human liver cancers. Hepatology. 2020;71:929–42.. doi: 10.1002/hep.30863. 31692012

[14] Chen CJ , Yang YH, Lin MH, et al. Herbal medicine containing aristolochic acid and the risk of primary liver cancer in patients with hepatitis C virus infection. Cancer Epidemiol Biomarkers Prev. 2019;28:1876–83.. doi: 10.1158/1055-9965.EPI-19-0023. 31409611

[15] Chen C , Yang Y, Lin M, et al. Herbal medicine containing aristolochic acid and the risk of hepatocellular carcinoma in patients with hepatitis B virus infection. Int J Cancer. 2018;143:1578–87.. doi: 10.1002/ijc.31544. 29667191

[16] Ng AWT , Poon SL, Huang M, et al. Aristolochic acids and their derivatives are widely implicated in liver cancers in Taiwan and throughout Asia. Sci Transl Med. 2017;9:eaan6446. doi: 10.1126/scitranslmed.aan6446. 29046434

[17] Healy ME , Chow JDY, Byrne FL, et al. Dietary effects on liver tumor burden in mice treated with the hepatocellular carcinogen diethylnitrosamine. J Hepatol. 2015;62:599–606.. doi: 10.1016/j.jhep.2014.10.024. 25450719 PMC4336610

[18] Stiborova M , Martã­Nek V, Frei E, et al. Enzymes metabolizing aristolochic acid and their contribution to the development of aristolochic acid nephropathy and urothelial cancer. Curr Drug Metab. 2013;14:695–705.. doi: 10.2174/1389200211314060006. 23701164

[19] Stiborovã¡ M , Arlt VM, Schmeiser HH. DNA Adducts Formed by Aristolochic Acid Are Unique Biomarkers of Exposure and Explain the Initiation Phase of Upper Urothelial Cancer. Int J Mol Sci. 2017;18:2144. doi: 10.3390/ijms18102144. 29036902 PMC5666826

[20] Pfau W , Schmeiser HH, Wiessler M. Aristolochic acid binds covalently to the exocyclic amino group of purine nucleotides in DNA. Carcinogenesis. 1990;11:313–9.. doi: 10.1093/carcin/11.2.313. 2302759

[21] Stiborovã¡ M , BÃ¡Rta FE, Levovã¡ KI, et al. A Mechanism of O-Demethylation of Aristolochic Acid I by Cytochromes P450 and Their Contributions to This Reaction in Human and Rat Livers: Experimental and Theoretical Approaches. Int J Mol Sci. 2015;16:27561–75.. doi: 10.3390/ijms161126047. 26593908 PMC4661905

[22] Zhang Q , Luo P, Chen J, et al. Dissection of targeting molecular mechanisms of aristolochic acid-induced nephrotoxicity via a combined deconvolution strategy of chemoproteomics and metabolomics. Int J Biol Sci. 2022;18:2003–17.. doi: 10.7150/ijbs.69618. 35342337 PMC8935225

[23] Ji H-J , Li J-Y, Wu S-F, et al. Two New Aristolochic Acid Analogues from the Roots of Aristolochia contorta with Significant Cytotoxic Activity. Molecules. 2020;26:44. doi: 10.3390/molecules26010044. 33374869 PMC7795626

[24] Jin Ke , Su K-K, Li T, et al. Hepatic premalignant alterations triggered by human nephrotoxin aristolochic acid I in canines. Cancer Prev Res (Phila). 2016;9:324–34.. doi: 10.1158/1940-6207.CAPR-15-0339. 26851235

[25] Li T , Jin Ke, Zhu D-Y, et al. Premalignant alteration assessment in liver-like tissue derived from embryonic stem cells by aristolochic acid I exposure. Oncotarget. 2016;7:78872–82.. doi: 10.18632/oncotarget.12424. 27713163 PMC5346684

[26] Ramachandran P , Matchett KP, Dobie R, et al. Single-cell technologies in hepatology: new insights into liver biology and disease pathogenesis. Nat Rev Gastroenterol Hepatol. 2020;17:457–72.. doi: 10.1038/s41575-020-0304-x. 32483353

[27] Saviano A , Henderson NC, Baumert TF. Single-cell genomics and spatial transcriptomics: Discovery of novel cell states and cellular interactions in liver physiology and disease biology. J Hepatol. 2020;73:1219–30.. doi: 10.1016/j.jhep.2020.06.004. 32534107 PMC7116221

[28] Zhang B , Dai Y, Zhu L, et al. Single-cell sequencing reveals novel mechanisms of Aflatoxin B1-induced hepatotoxicity in S phase-arrested L02 cells. Cell Biol Toxicol. 2020;36:603–8.. doi: 10.1007/s10565-020-09547-z. 32607778

[29] Umbaugh DS , Ramachandran A, Jaeschke H. Spatial reconstruction of the early hepatic transcriptomic landscape after an acetaminophen overdose using single-cell RNA-sequencing. Toxicol Sci. 2021;182:327–45.. doi: 10.1093/toxsci/kfab052. 33983442 PMC8331154

[30] Seki E , Schwabe RF. Hepatic inflammation and fibrosis: functional links and key pathways. Hepatology. 2015;61:1066–79.. doi: 10.1002/hep.27332. 25066777 PMC4306641

[31] Krenkel O , Hundertmark J, Ritz T, et al. Single cell RNA sequencing identifies subsets of hepatic stellate cells and myofibroblasts in liver fibrosis. Cells. 2019;8:503. doi: 10.3390/cells8050503. 31137713 PMC6562512

[32] Zheng C , Zheng L, Yoo J-K, et al. Landscape of infiltrating T cells in liver cancer revealed by single-cell sequencing. Cell. 2017;169:1342–56.. doi: 10.1016/j.cell.2017.05.035. 28622514

[33] Luo P , Zhang Q, Zhong T-Yu, et al. Celastrol mitigates inflammation in sepsis by inhibiting the PKM2-dependent Warburg effect. Military Medical Research. 2022;9:22. doi: 10.1186/s40779-022-00381-4. 35596191 PMC9121578

[34] Chen S , Zhou Y, Chen Y, et al. fastp: an ultra-fast all-in-one FASTQ preprocessor. Bioinformatics. 2018;34:i884–90.. doi: 10.1093/bioinformatics/bty560. 30423086 PMC6129281

[35] Yu G , Wang Li-G, Han Y, et al. clusterProfiler: an R package for comparing biological themes among gene clusters. Omics. 2012;16:284–7.. doi: 10.1089/omi.2011.0118. 22455463 PMC3339379

[36] Hzelmann S , Castelo R, Guinney J. GSVA: gene set variation analysis for microarray and RNA-seq data. BMC Bioinf. 2013;14:7. doi: 10.1186/1471-2105-14-7. PMC3618321 23323831

[37] Ritchie ME , Phipson B, Wu Di, et al. limma powers differential expression analyses for RNA-sequencing and microarray studies. Nucleic Acids Res. 2015;43:e47. doi: 10.1093/nar/gkv007. 25605792 PMC4402510

[38] Trapnell C , Cacchiarelli D, Grimsby J, et al. The dynamics and regulators of cell fate decisions are revealed by pseudotemporal ordering of single cells. Nat Biotechnol. 2014;32:381–6.. doi: 10.1038/nbt.2859. 24658644 PMC4122333

[39] Efremova M , Vento-Tormo M, Teichmann SA, et al. CellPhoneDB: inferring cell-cell communication from combined expression of multi-subunit ligand-receptor complexes. Nat Protoc. 2020;15:1484–506.. doi: 10.1038/s41596-020-0292-x. 32103204

[40] Fang Z-E , Wang C, Niu M, et al. Integration of transcriptomic and metabolomic data to compare the hepatotoxicity of neonatal and adult mice exposed to aristolochic acid I. Front Genet. 2022;13:840961. doi: 10.3389/fgene.2022.840961. 35401701 PMC8992794

[41] Su T , Yang Y, Lai S, et al. Single-cell transcriptomics reveals zone-specific alterations of liver sinusoidal endothelial cells in cirrhosis. Cell Mol Gastroenterol Hepatol. 2021;11:1139–61.. doi: 10.1016/j.jcmgh.2020.12.007. 33340713 PMC7903131

[42] Lacy P . Mechanisms of degranulation in neutrophils. Allergy Asthma Clin Immunol. 2006;2:98–108.. doi: 10.1186/1710-1492-2-3-98. 20525154 PMC2876182

[43] Lignitto L , Leboeuf SE, Homer H, et al. Nrf2 activation promotes lung cancer metastasis by inhibiting the degradation of Bach1. Cell. 2019;178:316–329.e318.. doi: 10.1016/j.cell.2019.06.003. 31257023 PMC6625921

[44] Breuer DA , Pacheco MC, Washington MK, et al. CD8(+) T cells regulate liver injury in obesity-related nonalcoholic fatty liver disease. Am J Physiol Gastrointest Liver Physiol. 2020;318:G211–g224.. doi: 10.1152/ajpgi.00040.2019. 31709830 PMC7052570

[45] Baudoux T , Husson C, De Prez E, et al. CD4(+) and CD8(+) T cells exert regulatory properties during experimental acute aristolochic acid nephropathy. Sci Rep. 2018;8:5334. doi: 10.1038/s41598-018-23565-2. 29593222 PMC5871862

[46] Appenzeller Herzog C , Hartleif S, Vionnet J. Clinical parameters and biomarkers predicting spontaneous operational tolerance after liver transplantation: A scoping review. Am J Transplant. 2021;21:3312–23.. doi: 10.1111/ajt.16585. 33783969

[47] Yunna C , Mengru Hu, Lei W, et al. Macrophage M1/M2 polarization. Eur J Pharmacol. 2020;877:173090. doi: 10.1016/j.ejphar.2020.173090. 32234529

[48] Bastek H , Zubel T, Stemmer K, et al. Comparison of aristolochic acid I derived DNA adduct levels in human renal toxicity models. Toxicology. 2019;420:29–38.. doi: 10.1016/j.tox.2019.03.013. 30940547

[49] Abdullah R , Alhusainy W, Woutersen J, et al. Predicting points of departure for risk assessment based on in vitro cytotoxicity data and physiologically based kinetic (PBK) modeling: The case of kidney toxicity induced by aristolochic acid I. Food Chem Toxicol. 2016;92:104–16.. doi: 10.1016/j.fct.2016.03.017. 27016491

[50] Chen J , Luo P, Wang C, et al. Integrated single-cell transcriptomics and proteomics reveal cellular-specific response and microenvironment remodeling in aristolochic acid nephropathy. JCI Insight. 2022;7:e157360. doi: 10.1172/jci.insight.157360. 35852860 PMC9462482

[51] Macparland SA , Liu JC, Ma X-Z, et al. Single cell RNA sequencing of human liver reveals distinct intrahepatic macrophage populations. Nat Commun. 2018;9:4383. doi: 10.1038/s41467-018-06318-7. 30348985 PMC6197289

[52] Halpern KB , Shenhav R, Matcovitch-Natan O, et al. Single-cell spatial reconstruction reveals global division of labour in the mammalian liver. Nature. 2017;542:352–6.. doi: 10.1038/nature21065. 28166538 PMC5321580

[53] Aizarani N , Saviano A, Sagar, et al. A human liver cell atlas reveals heterogeneity and epithelial progenitors. Nature. 2019;572:199–204.. doi: 10.1038/s41586-019-1373-2. 31292543 PMC6687507

[54] Tanaka M , Itoh T, Tanimizu N, et al. Liver stem/progenitor cells: their characteristics and regulatory mechanisms. J Biochem. 2011;149:231–9.. doi: 10.1093/jb/mvr001. 21217146

[55] Deng X , Zhang X, Li W, et al. Chronic liver injury induces conversion of biliary epithelial cells into hepatocytes. Cell Stem Cell. 2018;23:114–122.e113.. doi: 10.1016/j.stem.2018.05.022. 29937200

[56] Malhi H , Guicciardi ME, Gores GJ. Hepatocyte death: a clear and present danger. Physiol Rev. 2010;90:1165–94.. doi: 10.1152/physrev.00061.2009. 20664081 PMC2943859

[57] Marin V , Poulsen K, Odena G, et al. Hepatocyte-derived macrophage migration inhibitory factor mediates alcohol-induced liver injury in mice and patients. J Hepatol. 2017;67:1018–25.. doi: 10.1016/j.jhep.2017.06.014. 28647568 PMC5650516

[58] Arshad MI , Piquet-Pellorce C, L'helgoualc'h A, et al. TRAIL but not FasL and TNFα, regulates IL-33 expression in murine hepatocytes during acute hepatitis. Hepatology. 2012;56:2353–62. 22961755 10.1002/hep.25893

[59] Upton J-P , Austgen K, Nishino M, et al. Caspase-2 cleavage of BID is a critical apoptotic signal downstream of endoplasmic reticulum stress. Mol Cell Biol. 2008;28:3943–51.. doi: 10.1128/MCB.00013-08. 18426910 PMC2423129

[60] Micheau O , Tschopp J. Induction of TNF receptor I-mediated apoptosis via two sequential signaling complexes. Cell. 2003;114:181–90.. doi: 10.1016/s0092-8674(03)00521-x. 12887920

[61] Ding Bi-S , Nolan DJ, Butler JM, et al. Inductive angiocrine signals from sinusoidal endothelium are required for liver regeneration. Nature. 2010;468:310–5.. doi: 10.1038/nature09493. 21068842 PMC3058628

[62] Iwakiri Y . Endothelial dysfunction in the regulation of cirrhosis and portal hypertension. Liver Int. 2012;32:199–213.. doi: 10.1111/j.1478-3231.2011.02579.x. 21745318 PMC3676636

[63] Thabut D , Shah V. Intrahepatic angiogenesis and sinusoidal remodeling in chronic liver disease: new targets for the treatment of portal hypertension?. J Hepatol. 2010;53:976–80.. doi: 10.1016/j.jhep.2010.07.004. 20800926

[64] Deleve L . Hepatic microvasculature in liver injury. Semin Liver Dis. 2007;27:390–400.. doi: 10.1055/s-2007-991515. 17979075

[65] Karaa A , Kamoun WS, Clemens MG. Oxidative stress disrupts nitric oxide synthase activation in liver endothelial cells. Free Radical Biol Med. 2005, 39:1320–31.. doi: 10.1016/j.freeradbiomed.2005.06.014. 16257641

[66] Raquel GB, Arnau PR, Sergio SN, et al. Nrf2 and oxidative stress in liver ischemia/reperfusion injury. FEBS J. 2021;289:5463–79.. doi: 10.1111/febs.16336.

[67] Zhao J , Qi Y-F, Yu Y-R. STAT3: A key regulator in liver fibrosis. Ann Hepatol. 2021;21:100224. doi: 10.1016/j.aohep.2020.06.010. 32702499

[68] Ji H , Hu J, Zhang G, et al. Aristolochic acid nephropathy: A scientometric analysis of literature published from 1971 to 2019. Medicine (Baltimore). 2021;100:e26510. doi: 10.1097/MD.0000000000026510. 34232183 PMC8270620

[69] Tacke F , Zimmermann HW. Macrophage heterogeneity in liver injury and fibrosis. J Hepatol. 2014;60:1090–6.. doi: 10.1016/j.jhep.2013.12.025. 24412603

[70] Wan J , Benkdane M, Teixeira-Clerc F, et al. M2 Kupffer cells promote M1 Kupffer cell apoptosis: a protective mechanism against alcoholic and nonalcoholic fatty liver disease. Hepatology. 2014;59:130–42.. doi: 10.1002/hep.26607. 23832548

[71] Sadatomo Ai , Inoue Y, Ito H, et al. Interaction of neutrophils with macrophages promotes IL-1β maturation and contributes to hepatic ischemia-reperfusion injury. J Immunol. 2017;199:3306–15.. doi: 10.4049/jimmunol.1700717. 28972095

[72] Elshal M , Abdelmageed ME. Diacerein counteracts acetaminophen-induced hepatotoxicity in mice via targeting NLRP3/caspase-1/IL-1β and IL-4/MCP-1 signaling pathways. Arch Pharmacal Res. 2022;45:142–58.. doi: 10.1007/s12272-022-01373-7. PMC8967791 35244883

[73] Yan T , Yan N, Wang H, et al. FXR-deoxycholic acid-TNF-α axis modulates acetaminophen-induced hepatotoxicity. Toxicol Sci. 2021;181:273–84.. doi: 10.1093/toxsci/kfab027. 33662127 PMC8163055

[74] Wang Y , Zhang H, Chen Q, et al. TNF-α/HMGB1 inflammation signalling pathway regulates pyroptosis during liver failure and acute kidney injury. Cell Prolif. 2020;53:e12829. doi: 10.1111/cpr.12829. 32419317 PMC7309595

[75] Lu H , Bai Y, Wu L, et al. Inhibition of macrophage migration inhibitory factor protects against inflammation and matrix deposition in kidney tissues after injury. Mediators Inflamm. 2016;2016:2174682. doi: 10.1155/2016/2174682. 27313397 PMC4893598

[76] Honarpisheh M , Foresto-Neto O, Steiger S, et al. Aristolochic acid I determine the phenotype and activation of macrophages in acute and chronic kidney disease. Sci Rep. 2018;8:12169. doi: 10.1038/s41598-018-30628-x. 30111809 PMC6093867

[77] Wang Y , Liu Y. Neutrophil-induced liver injury and interactions between neutrophils and liver sinusoidal endothelial cells. Inflammation. 2021;44:1246–62.. doi: 10.1007/s10753-021-01442-x. 33649876

