# Supplementary material for: Dissection of cellular and molecular mechanisms of aristolochic acid-induced hepatotoxicity via single-cell transcriptomics
Source: Precis Clin Med. 2022 Sep 22;5(4):pbac023. doi: 10.1093/pcmedi/pbac023 (PMC9635452; doi:10.1093/pcmedi/pbac023)
Supplement: pbac023_Supplemental_File [file pbac023_supplemental_file.docx]

**Supplementary materials**


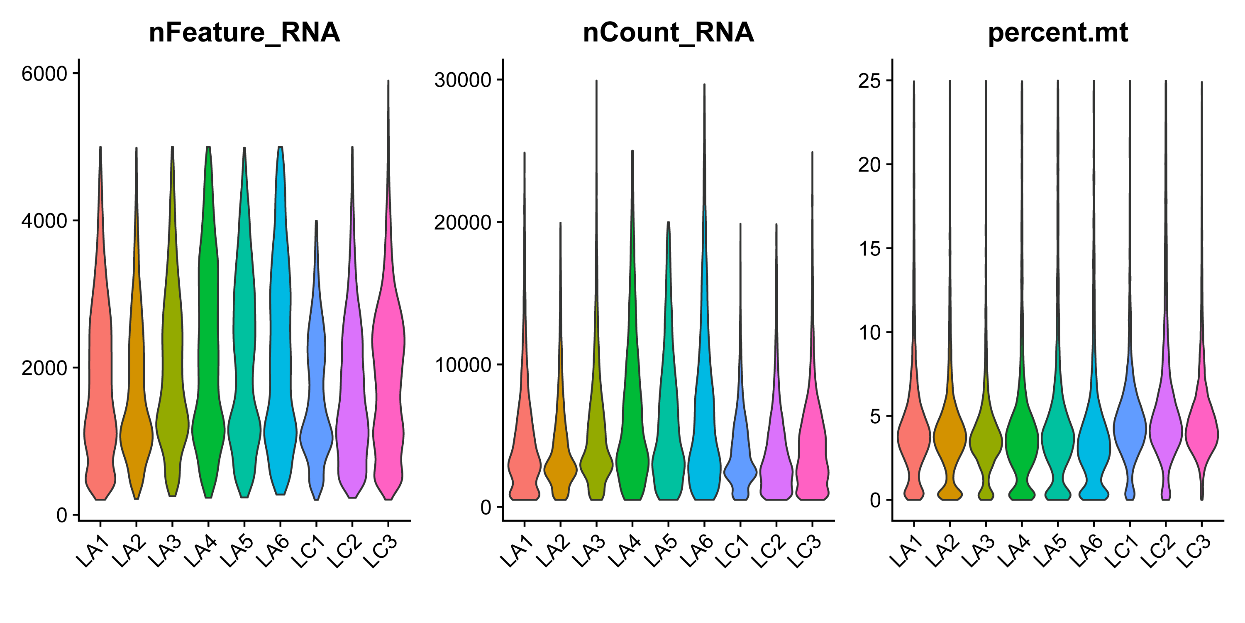


**Figure. S1.** The violin plot shows the relative level of RNA feature number, RNA counts number and mitochondria gene percent across 9 samples after quality control in scRNA-seq datasets.


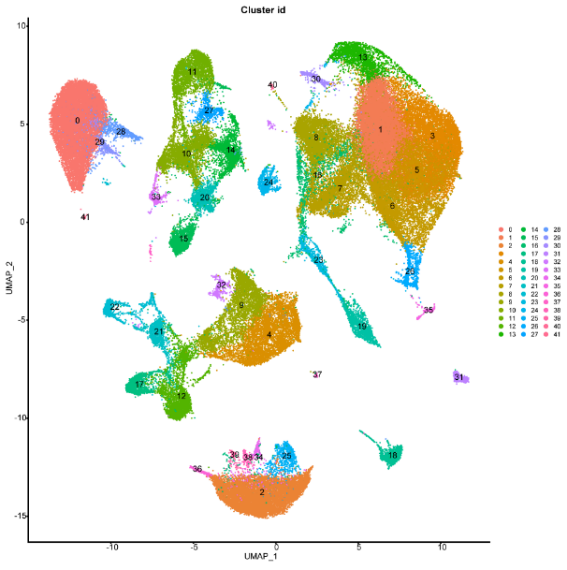


**Figure. S2.** The UMAP visualization shows unsupervised single-cell transcriptome 42 clusters of integrated datasets.


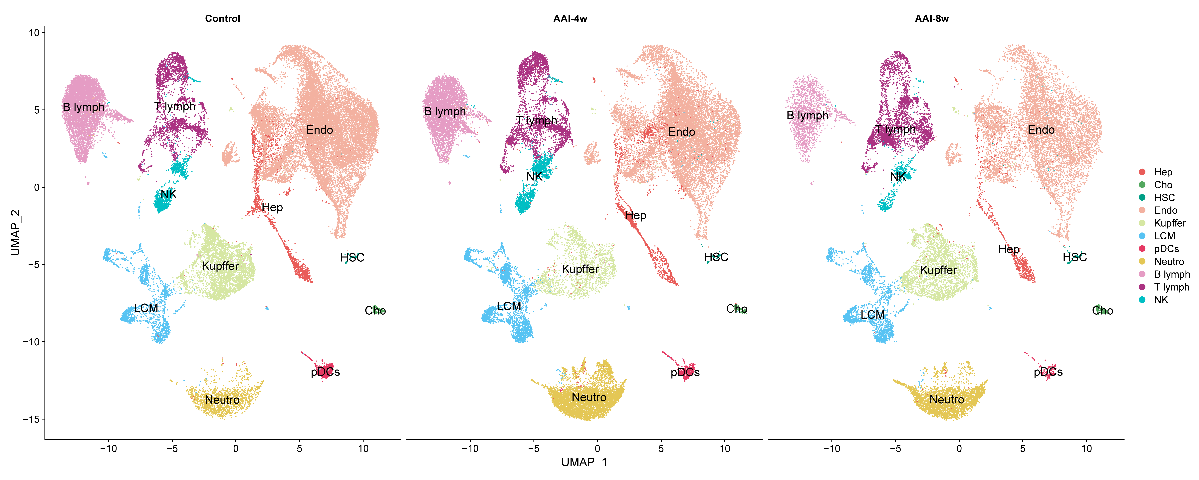


**Figure. S3.** The UMAP visualization shows unsupervised single-cell transcriptome clustering split into control, AAI-4w and AAI-8w groups, revealing 11 major cell types based on the relative expressions of canonical markers in mouse liver.


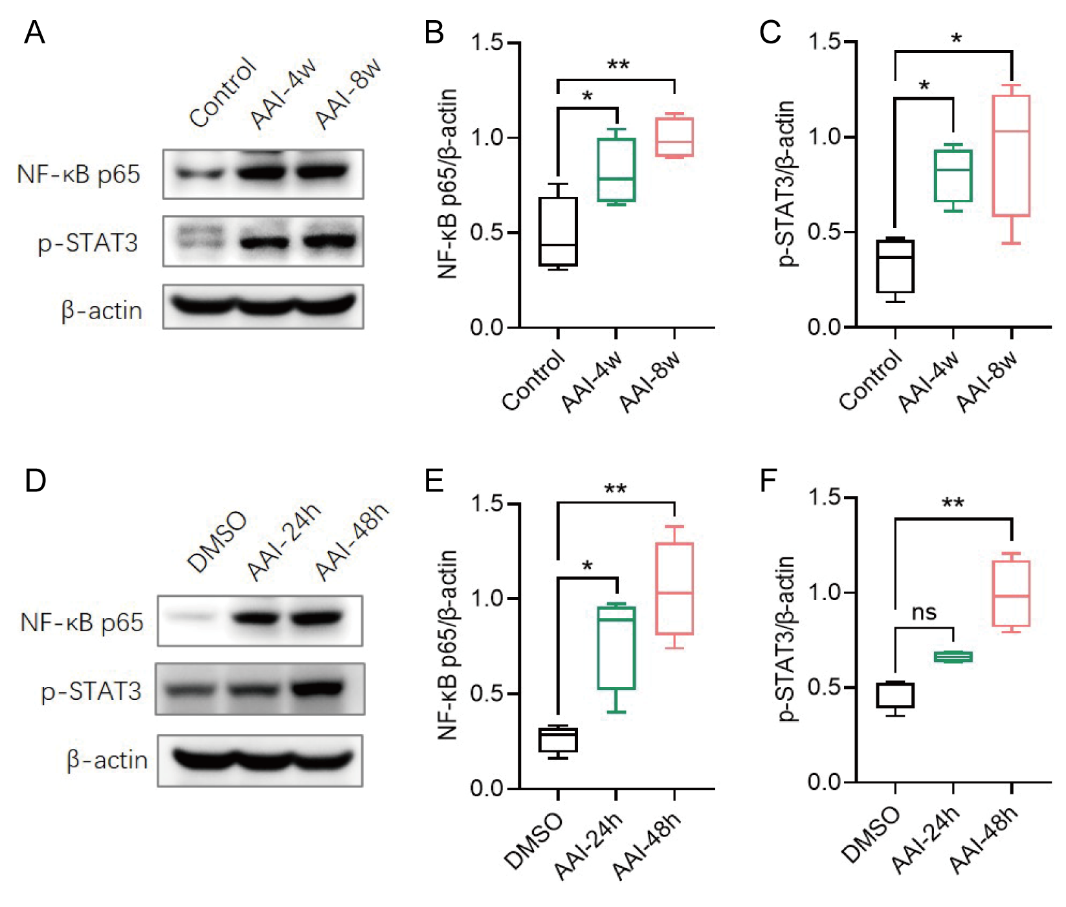


**Figure. S4**. Western blotting analysis of the activation of STAT3 and NF-κB in vivo and in vitro. (A-C) Western blot assay to analyze the STAT3 phosphorylation and NF-κB p65 expression in the liver of mice with or without AAI treatment (n = 4, *P < 0.05, **P < 0.01 vs Control). (D-F) Western blot assay to analyze the STAT3 phosphorylation and NF-κB p65 expression in hepatocytes (NCTC 1469 cell) with or without AAI treatment (n = 4, **P* < 0.05, ***P* < 0.01 vs DMSO; ns, no significance).


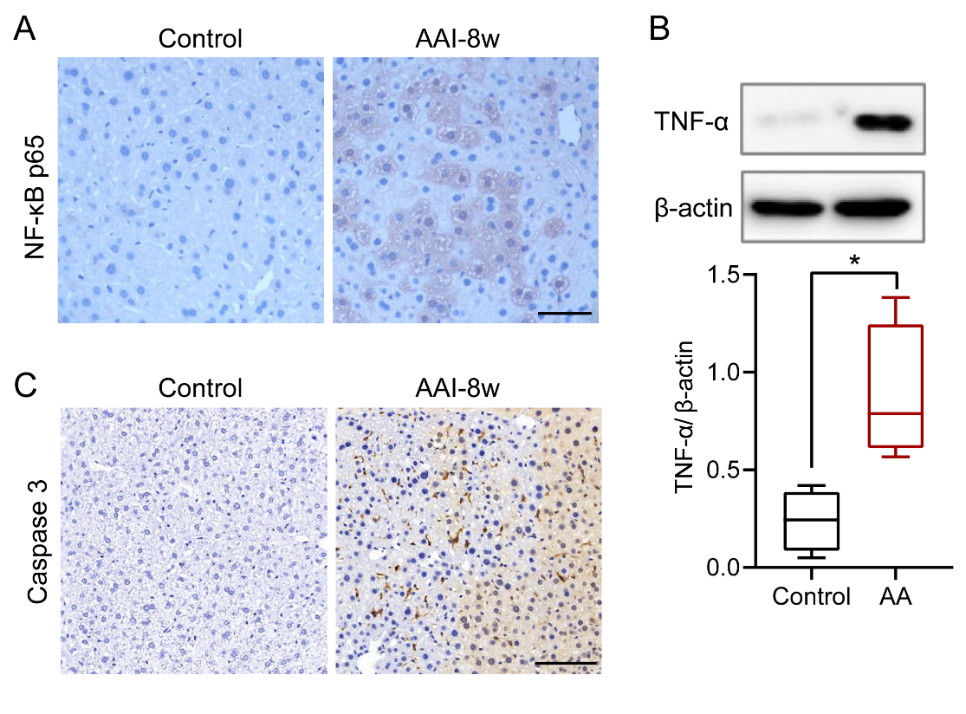


**Figure. S5. AAI induces hepatocyte cells apoptosis via inflammation response.** (A) Immunohistochemistry staining of NF-κB p65 in liver, scale bar = 100 μm. (B) Western blot assay to analyze the TNF-α expression in NCTC 1469 cell with or without AAI treatment, (n = 4, **P* < 0.05 vs Control). (C) Immunohistochemistry staining to investigate the effect of AAI on Caspase 3 expression, scale bar = 150 μm.


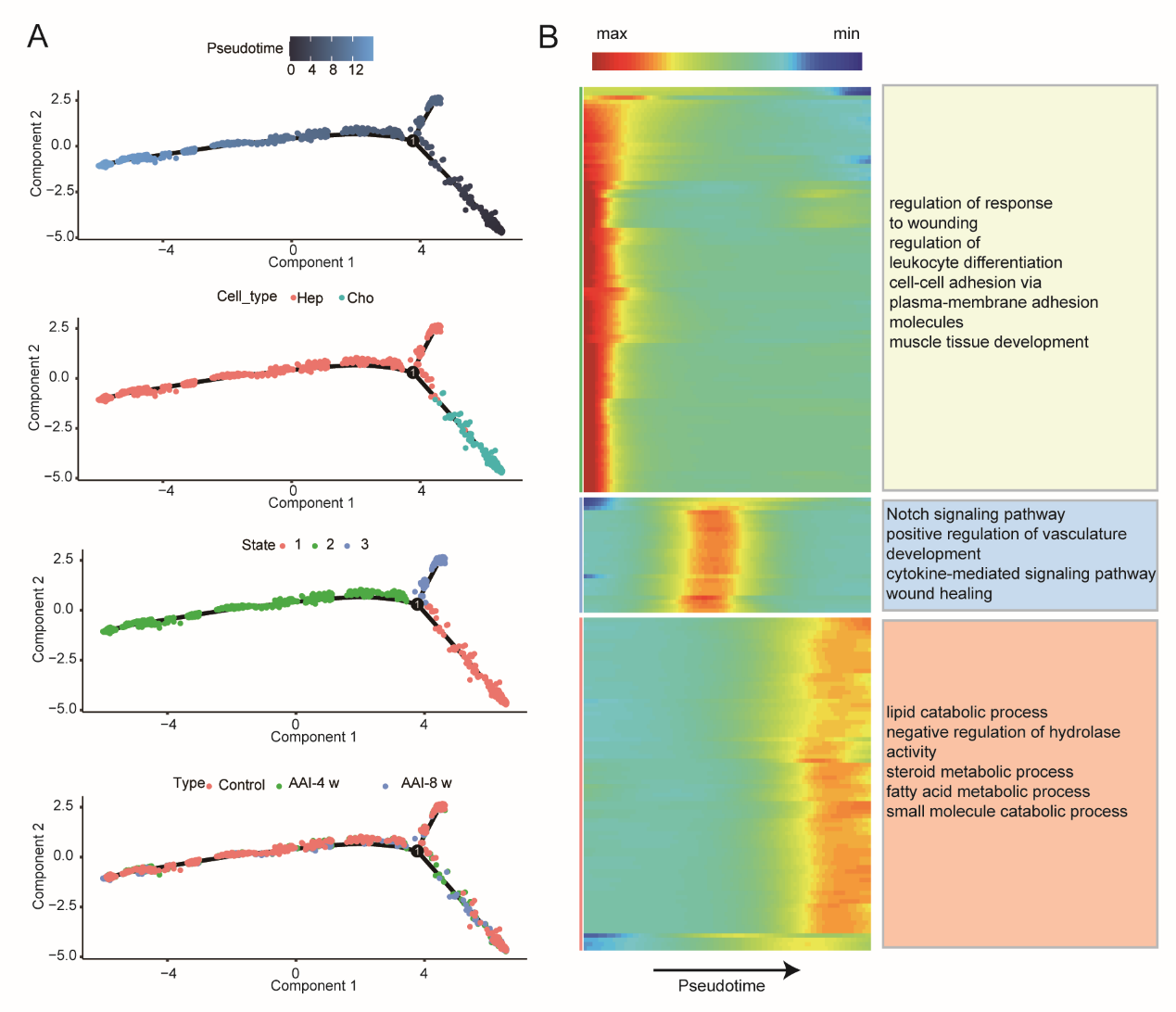


**Figure. S6.** The pesudotime path analysis of the transdifferentiation of cholangiocytes into hepatocytes. (A) Monocle trajectory inference of cholangiocytes traces the path of pesudotime, cell types, label with the cell state, and sample group ID from top to bottom, respectively. (B) The heatmap plot reveals the relative gene expression level of clusters based on branched expression analysis modeling, combined with the GO enriched items of each cluster after AAI treatment, the pesudotime from left to right.


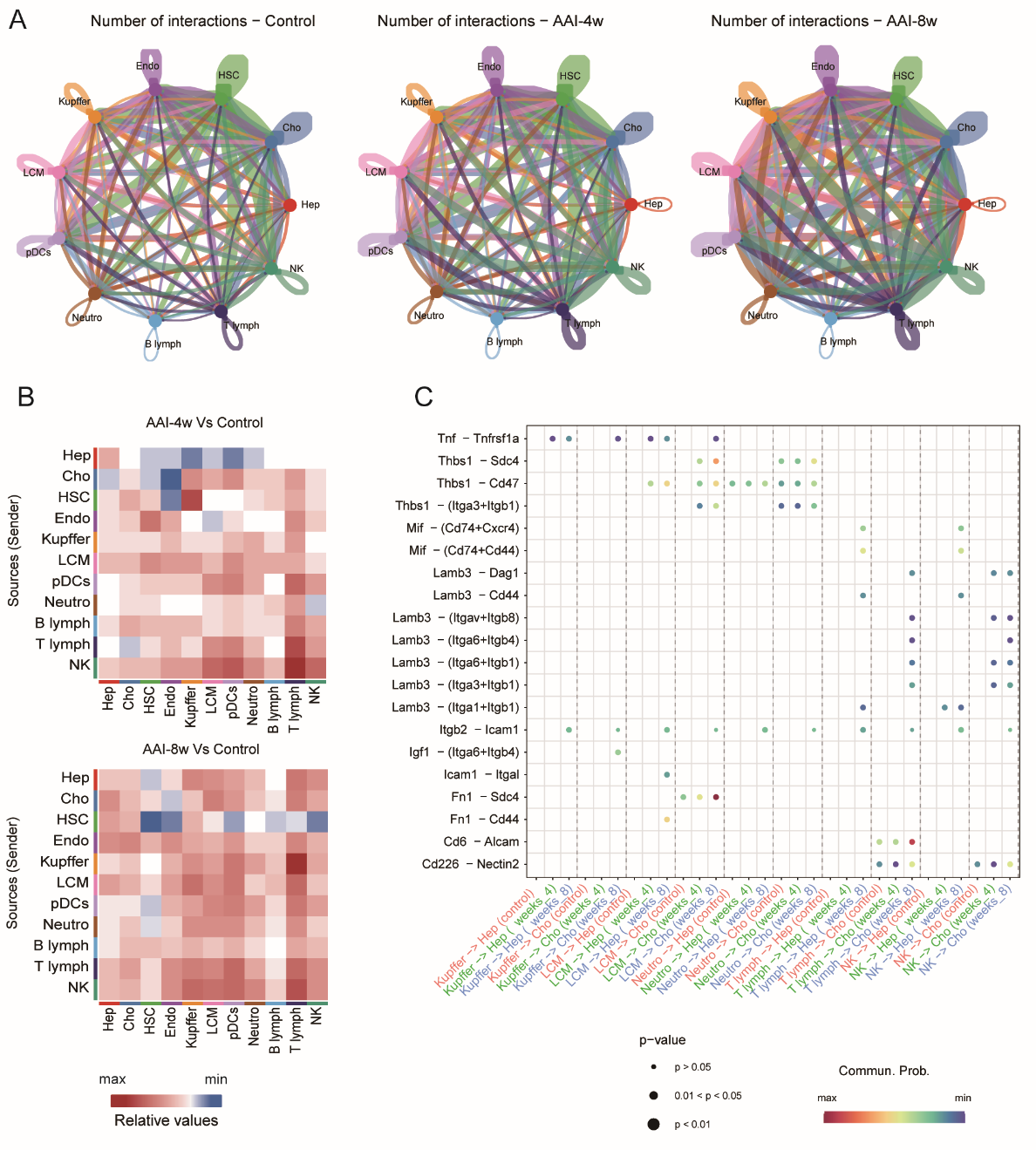


**Figure. S7.** Intercellular networks for the response to AAI in liver microenvironment. (A) The chordal graph of total cell to cell interaction number of cell types among Control, AAI-4w and AAI-8w groups, colored according to each cell type, the thickness degree indicates the interaction strength between sender and receiver cell. (B) The heatmap plot shows the differential interaction numbers between the sender and receiver subtypes cell in the AAI-4w or AAI-8w group compared to Control group. The top bar plot represents the sum of incoming signaling and the right represents the sum of outgoing signaling. (C) The bobble plot shows significant up-regulated ligand-receptor pairs between sender and receiver cell, colored according to group types.
